# Self-assembled fatty acid crystalline coatings display superhydrophobic antimicrobial properties

**DOI:** 10.1016/j.mtbio.2022.100516

**Published:** 2022-12-08

**Authors:** Elena Prudnikov, Iryna Polishchuk, Andy Sand, Hanan Abu Hamad, Naama Massad-Ivanir, Ester Segal, Boaz Pokroy

**Affiliations:** a Department of Materials Science and Engineering, Technion − Israel Institute of Technology, 3200003 Haifa, Israel; b Faculty of Biotechnology and Food Engineering, Technion − Israel Institute of Technology, 3200003 Haifa, Israel

**Keywords:** Superhydrophobic coatings, Saturated fatty acids, Thermal deposition, Spray coating, Antimicrobial, Anti-biofouling, Biocidal, CA, contact angle, CAH, contact angle hysteresis, SFA, saturated fatty acid

## Abstract

Superhydrophobicity is a well-known wetting phenomenon found in numerous plants and insects. It is achieved by the combination of the surface's chemical properties and its surface roughness. Inspired by nature, numerous synthetic superhydrophobic surfaces have been developed for various applications. Designated surface coating is one of the fabrication routes to achieve the superhydrophobicity. Yet, many of these coatings, such as fluorine-based formulations, may pose severe health and environmental risks, limiting their applicability. Herein, we present a new family of superhydrophobic coatings comprised of natural saturated fatty acids, which are not only a part of our daily diet, but can be produced from renewable feedstock, providing a safe and sustainable alternative to the existing state-of-the-art. These crystalline coatings are readily fabricated via single-step deposition routes, namely thermal deposition or spray-coating. The fatty acids self-assemble into highly hierarchical crystalline structures exhibiting a water contact angle of ∼165° and contact angle hysteresis lower than 6°, while their properties and morphology depend on the specific fatty acid used as well as on the deposition technique. Moreover, the fatty acid coatings demonstrate excellent thermal stability. Importantly, this new family of coatings displays excellent anti-biofouling and antimicrobial properties against *Escherichia coli* and *Listeria innocua*, used as relevant model Gram-negative and Gram-positive bacteria, respectively. These multifunctional coatings hold immense potential for application in numerous fields, ranging from food safety to biomedicine, offering sustainable and safe solutions.

## Graphical abstract

**Figure:**
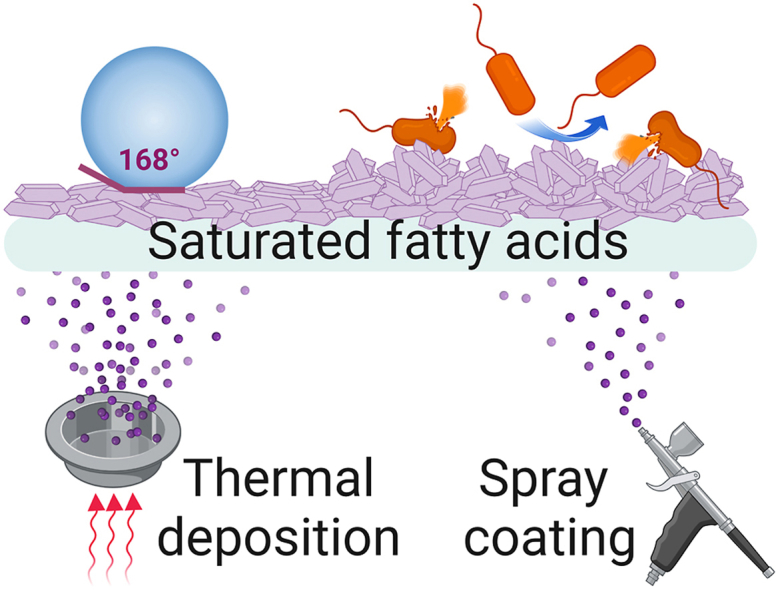

## 1 Introduction

Nature is replete with materials that demonstrate unique functional features such as optical and magnetic properties for sensing, mechanical properties for improved strength, superhydrophobicity for self-cleaning and more [[1], [2], [3], [4], [5]]. These properties are achieved due to exceptional balance of the structure-function relationship [6,7].

The phenomenon of superhydrophobicity is well known for many years now and is commonly observed in a wide range of organisms like plants and insects [[7], [8], [9]]. Superhydrophobicity often provides organisms with additional functional properties such as self-cleaning, enhanced flight capability, thermal isolation and sensory capabilities [7,8]. Superhydrophobic surfaces demonstrate water contact angles (CA) higher than 150°, and contact angle hysteresis (CAH) lower than 10° in the case that the surface exhibits self-cleaning properties as well. The surface wetting can occur under 3 states: Young state, which describes a droplet on a flat surface [10]; Wenzel state, which describes a wetting of rough surface with homogeneous solid-liquid interface [11]; Cassie-Baxter state, which describes a condition when the droplet is only partially supported by the rough surface, while free liquid surface is found in between the supporting points [12]. Cassie-Baxter state, which assures superhydrophobicity is achieved by the combination of intrinsic hydrophobicity of the surface and its appropriate roughness. Moreover, hierarchical structure of the surface greatly contributes to lowering the surface-liquid contact area, thereby increasing CA values [10,12,13]. Superhydrophobic surfaces are also interesting as bio-inspired artificial analogues which exhibit other functional surface properties such as water repellence, anti-fog, anti-icing, reduced adhesion, anti-corrosion, antibiofouling and antimicrobial properties [7,[14], [15], [16], [17], [18], [19], [20], [21]].

Multiple approaches have been employed to design superhydrophobic surfaces demonstrating a necessary combination of the appropriate roughness and chemical properties such as lithography and templating methods, plasma treatments, electrochemical processes, various deposition methods, as well as completely different strategy based on slippery liquid-infused porous surfaces (SLIPS) [[22], [23], [24], [25], [26], [27], [28]]. Yet, most of these fabrication routes are complicated, expensive or limited to specific materials and may be difficult for scaling.

Previously, we have demonstrated a bio-inspired approach to form self-cleaning superhydrophobic surfaces composed of paraffin wax crystals, which self-assemble into highly-oriented hierarchical structures thereby forming a superhydrophobic surface coating. These coatings were shown to be applied onto various types of surfaces via thermal deposition [[29], [30], [31]]. Time-dependent tuning of the coating's wetting properties and dependence of the superhydrophobic performance on molecular weight of the various wax crystals or their combination were studied [29,32]. Moreover, we have demonstrated that these bio-inspired wax coatings, in particular those composed of fluorinated waxes, exhibit prominent antibiofouling properties achieved by their ability to passively eliminate bacterial attachment to the coated surfaces [33]. These properties were also harnessed for preventing biofilm establishment in challenging environments, such as dairy storage and production [34]. Other studies showed efficient antifungal activity of the paraffin-based paper with incorporated essential oils [35]. Similar approach employing incorporation of active antimicrobials is used for gelatin-based films [36]. However, in these cases the antimicrobial effect is mainly attributed to the additives rather than to the matrix's material.

In the current study we aimed to form multifunctional superhydrophobic coatings comprised of non-toxic saturated fatty acids (SFAs). These molecules are naturally present in biological systems, including human body, and are a part of human daily dietary uptake [[37], [38], [39], [40]]. The latter makes fatty acids promising candidate compounds to serve as a coating agent for various applications where strict safety regulations applies, such as food contact surfaces, agricultural, biomedical etc. [[41], [42], [43]] In contrast to paraffin waxes, fatty acids contain a terminal carboxylic group, which may be involved in their antibacterial activity [44] and can, therefore, affect the coating's properties as well. Moreover, fatty acids are known as natural antimicrobial agents, which makes them even more advantageous for application in functional coatings due to the synergetic effect of their intrinsic superhydrophobic and antimicrobial properties [44,45].

Several previous studies have demonstrated the usage of SFAs as attractive components in various coating formulations. For example, coatings containing stearic acid or lauric acid were proposed for extending the shelf life of apple slices [46] and beef [47], respectively, owing to the intrinsic antimicrobial and antioxidative properties of SFAs. Ivanova et al. have demonstrated the bactericidal activity of self-assembled palmitic and stearic acid films when recrystallized on highly ordered pyrolytic graphite substrates [48]. Superhydrophobic surfaces were also achieved via self-assembly of multi-component solid and liquid crystal materials, where fatty acids serve as a component in the coating precursor, and their functionality strongly depended on additional components [[49], [50], [51]]. Moreover, in the reported case of stearic acid, its using as a single component did not resulte in superhydrophobic properties of the surface [50]. Applying to the surface SiO_2_ ​particles ​pre-coated with long carbon chain fatty acids was also suggested to form superhydrophobic coatings [52]. However, the mentioned studies could not achieve multifunctional superhydrophobic coatings using a single-component SFA formulations.

Herein, we present facile deposition methods to form superhydrophobic coatings using various SFAs. Resulted coatings were characterized in order to investigate their physical, crystallographic, structural and thermal stability properties. We also demonstrate the effect of the molecular length of SFAs and the effect of different deposition techniques on the coating's properties. In addition, the anti-biofouling and antimicrobial activities of the spray-deposited coatings were examined against *Escherichia coli* (*E. coli*) and *Listeria innocua* (*L. innocua*), as relevant model bacteria.

## 2 Results and discussion

Based on our previous studies on thermally deposited paraffin wax coatings, we, firstly, studied the feasibility of forming fatty acids coatings via thermal deposition method. To this end, a series of six SFAs was selected: palmitic acid (16C) – 16 carbons, stearic acid (18C) – 18 carbons, arachidic acid (20C) – 20 carbons, behenic acid (22C) – 22 carbons, lignoceric acid (24C) – 24 carbons and cerotic acid (26C) – 26 carbons. Selected SFAs were thermally deposited on glass microscope slides (see Experimental section) using an identical amount of 125 ​± ​1 ​mg. In order to study the thermal stability of the SFA coatings, additional series of equivalent coatings were heated at 50 °C for 24 h in air. The properties of both as-deposited and post-heated coatings were further studied as a function of the carbon chain length of selected SFAs.

**Fig. 1:**
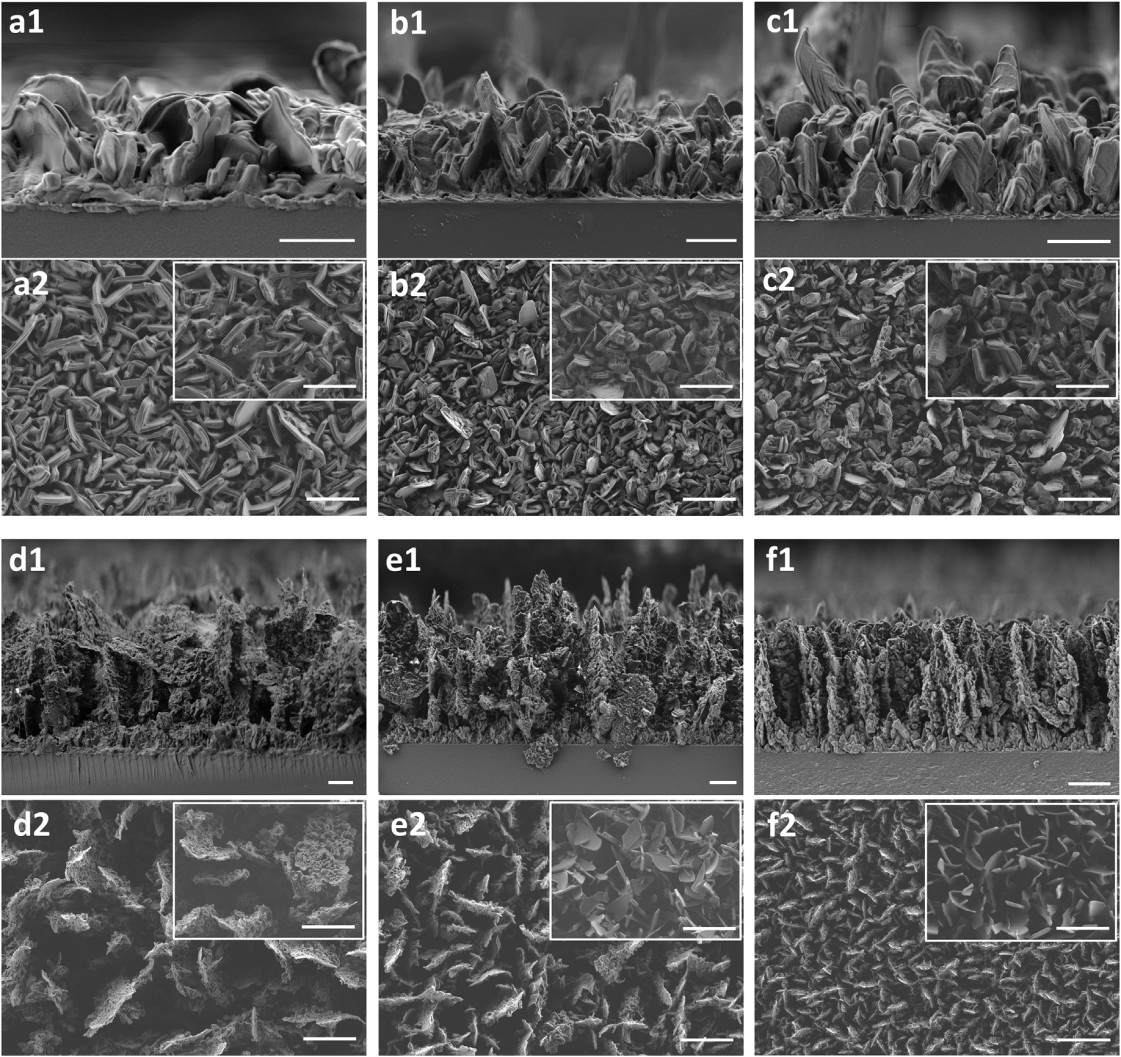
HR-SEM images of the deposited coatings before and after heating. Cross-sectional views of the coatings: a1) palmitic acid (16C), b1) stearic acid (18C), c1) arachidic acid (20C), d1) behenic acid (22C), e1) lignoceric acid (24C), f1) cerotic acid (26C). Scale bar is 2 ​μm a2-f2) Planar views of the as deposited coatings, respectively. Scale bar is 4 ​μm. Insets – planar view of the coatings treated thermally for 24 ​h at 50 ​°C, respectively. Scale bar is 4 ​μm.

The morphology of the resulted coatings was observed in cross-sectional and planar views acquired using a HR-SEM. As can be seen in Fig. 1, all the SFA coatings appear as a dense uniform assembly of crystals with a characteristic morphology. Moreover, their surface morphology imaged via planar views (Fig. 1 a1-f1) is also recognized across the whole depth of the coatings observed in corresponding cross-sectional views (Fig. 1 a2-f2). Following a detailed analysis, we could distinguish two types of coatings: group A - SFAs with 16–20 carbons, including palmitic, stearic and arachidic acids; and group B – SFAs with 22–26 carbons, including behenic, lignoceric and cerotic acids. The coatings from the group A comprise well-defined and edged crystals ∼2 ​μm in length and ∼0.5 ​μm in thickness (Fig. 1 a2-c2), while the crystals in the group B have smoother shape, showing a clear perpendicular orientation relative to the substrate. The backbone large crystals (2–6 ​μm) in group B are covered with smaller crystals up to few tens of nanometers in size (Fig. 1 d2-f2), thereby developing a hierarchical structure. In the group A of SFAs the crystals' size is not significantly affected by the carbon chain length of fatty acids, which is proportional to the number of carbons in the molecule, (Fig. 1 a2-c2). In contrast, the coatings in the group B show a clear correlation between the size of the crystals and the carbon chain length of a fatty acid. The longer the fatty acid, the smaller the obtained crystals: behenic acid (22C) crystals are ∼6 ​μm in length, lignoceric acid (24C) crystals are ∼4 ​μm in length and cerotic acid crystals are ∼2 ​μm in length (Fig. 1 d2-f2). It can also be seen in the cross-section views that the crystals of cerotic acid (26C) are better ordered than those of lignoceric acid (24C) and behenic acid (26C); this effect is probably achieved due to a smaller size of cerotic acid (26C) crystals, which form denser and thinner coating, therefore less degrees of freedom in growth orientation exist. The coarse characteristic morphology of SFA coatings from both groups can be attributed to the effect of the high aspect ratio of the molecules. This dimensional difference determines the crystals growth direction and results in the formation of anisotropic crystals comprising a rough surface.

The insets in Fig. 1 a2-f2 show the morphology of each coating after heat treatment. While no significant change could be noticed in the case of the most of the fatty acid coatings, the coatings formed by lignoceric acid (24C) and cerotic acid (26C) underwent significant morphological transformation. In particular, the nanometric crystals, covering the backbone crystals prior to the heat treatment, disappeared (Fig. 1 e2-f2, insets). Interestingly, the morphology was changed in the case of the fatty acids with the high melting points of 75–88 ​°C compared to melting points of unchanged coatings of SFAs – 63–80 ​°C (Table S1), even though they were expected to be more thermally stable at a given temperature.

**Fig. 2:**
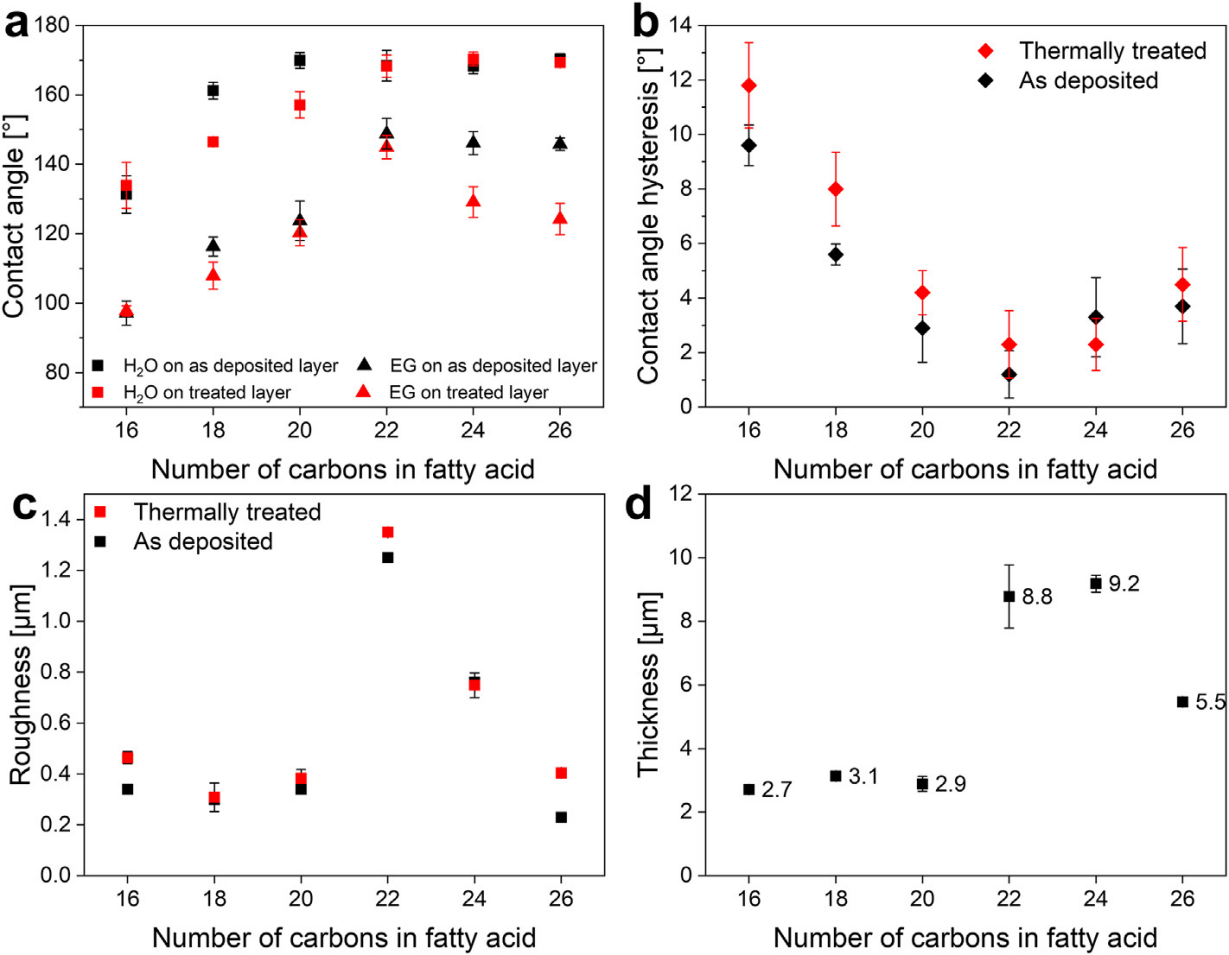
Characteristic properties of deposited fatty acid coatings as a function of carbons number in the molecule. Thermally treated coatings are presented by the red color. a) CA of water and ethylene glycol on the as-deposited coatings (black squares and triangles) and on the thermally treated coatings (red squares and triangles). b) Water CAH on the as-deposited coatings (black rhombi) and thermally treated coatings (red rhombi). c) Roughness of as-deposited coatings (black squares) and thermally treated coatings (red squares). d) Coating thickness of as-deposited coatings. Error bars correspond to standard deviation.

Wetting properties of the coatings before and after heating were studied via CA and CAH measurements (Fig. 2 a and b). As can be seen in Fig. 2a, as-deposited coatings composed of SFAs with 18–26 carbons demonstrated superhydrophobic behavior resulting in water (high surface tension [53] – 72.8 ​mN ​m^−2^) ​CA higher than 150°. Water CA increases up to ​∼170° as the carbon chain length extends from 16 to 20 carbonsand remains stable for longer molecules from group B. Such behavior is similar for both as-deposited and heat-treated samples. Similar trend is seen in the case of ethylene glycol (lower surface tension [53] – 48 ​mN ​m^−2^), however, its contact angle on post-heated lignoceric acid (24C) and cerotic acid (26C) coatings decreases (Fig. 2a, red triangles), probably as a result of the change in crystal's morphology observed after heat treatment (Fig. 1, a2-f2, insets). CAH results corroborate the CA values measured as a function of the number of carbons in a SFA (Fig. 2b): the higher the CA, the lower the CAH. Typically, low CAH values can be achieved on hierarchical superhydrophobic surfaces with good surface uniformity [[54], [55], [56]]. Therefore, the opposite trend of CAH values relatively to CA values of different coatings, combined with their morphology and the developed surface hierarchy of each coating was expected. The increase in CAH values in the case of the most samples after heating may indicate a higher surface heterogeneity caused by heat-induced crystal coarsening (Fig. 1, a2-c2 insets).

The roughness and thickness of the studied coatings were measured by means of a confocal microscope. The coatings from the group A with 16–20 carbons demonstrated similar roughness values ranging between 0.31 and 0.34 ​μm (Fig. 2c) as well as similar thicknesses of ∼3 ​μm (Fig. 2d). In the case of the coatings from the group B, the highest roughness of ∼1.3 ​μm is observed in the case of behenic acid (22C) coating, which can be explained by the bigger size of the crystals as seen in the HR-SEM images (see Fig. 1, d2). Lignoceric acid (24C) and cerotic acid (26C) demonstrate a roughness of ∼0.8 ​μm and ∼0.2 ​μm, respectively. However, behenic acid (22C) and lignoceric acid (24C) coatings are of similar thicknesses of ∼9 ​μm, while cerotic acid (26C) coating was measured to be ∼6 ​μm thick, which is a third less than that of the SFAs in group B. The relatively low values of roughness and thickness can be ascribed to a smaller size and denser packing of the crystals comprising cerotic acid (26C) coatings as was observed in HR-SEM images (Fig. 1, f1, f2). We also note that, thermal treatment had no significant effect on the roughness of all the SFAs coatings (Fig. 2c, red squares).

**Fig. 3:**
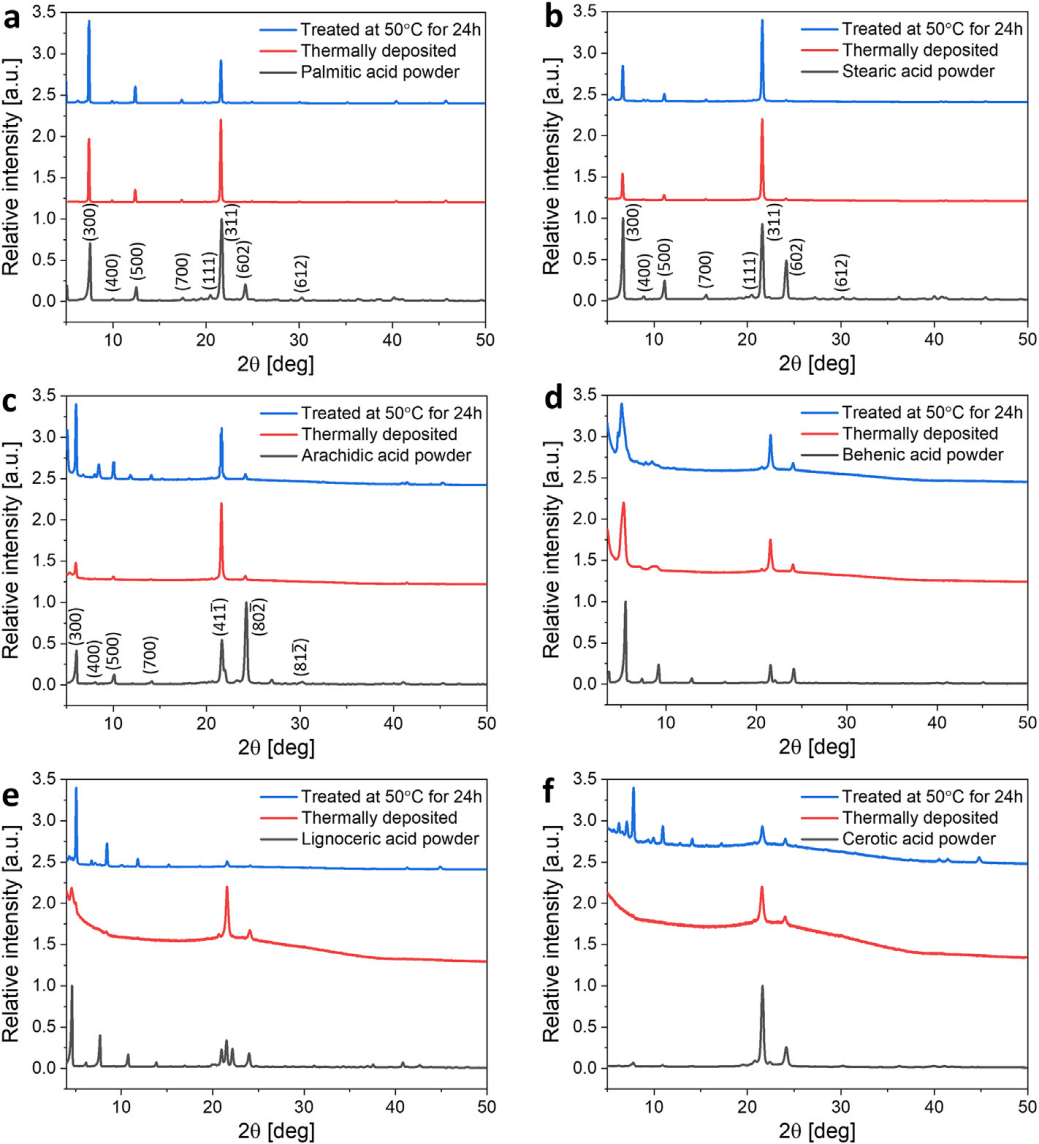
X-ray diffraction patterns of powdered fatty acids (black lines), as-deposited coatings (red lines) and thermally treated coatings (blue lines). a) Palmitic acid (16C), b) Stearic acid (18C), c) Arachidic acid (20C), d) Behenic acid (22C), e) Lignoceric acid (24C), f) Cerotic acid (26C).

In order to determine the crystal structure and crystallographic orientation of the as-deposited and heated coatings we collected X-ray diffraction patterns from the coatings and corresponding SFAs in a powdered form (Fig. 3). The diffraction patterns collected from the coatings of the group A were indexed according to the existing literature [57]. As evidenced by the diffraction patterns, SFA coatings included into the group A are highly crystalline with a clear preferred crystal orientation (Fig. 3, a-c, red lines). Palmitic acid (16C) and stearic acid (18C) coatings (Fig. 3, a-b) demonstrate a degree of (311) plane preferred orientation with *η* (preferred orientation degree) of 47% and 45%, respectively, which is calculated relative to the (602) plane (for details see Experimental section). Obtained rather low *η* values indicate the presence of a high preferred orientation since the angle between the mentioned above planes is relatively high (55.99° and 55.92°, respectively). In the case of arachidic acid (20C) coating, a $\left(41\bar{1}\right)$ preferred orientation is clearly seen (Fig. 3c). Analysis relative to the $\left(80\bar{2}\right)$ plane of its powdered sample results in *η* of 43% with an angle of 55.93° between the corresponding planes. The preferred orientation of the post-heated coatings was well maintained; minor changes in relative intensities of the diffraction peaks were observed after heat treatment of the coatings from the group A (Fig. 3, a-c, blue lines). Additional diffraction peaks of the {h00} family emerged only in the case of the heat-treated arachidic acid (20C) coating (Fig. 3c).

The crystallinity of the SFAs coatings from the group B decreased with an increase in the number of carbons of the fatty acids (Fig. 3, d-f). A characteristic amorphous hump is most prominent in the diffraction pattern of the cerotic acid (26C) coating. We assume that, the longer the SFA molecule, the higher is the diffusional barrier for crystallization due to the higher molecular weight, which reduces the diffusion coefficient. Therefore, more material is deposited in the amorphous state. The diffraction patterns of the group B were not indexed since no crystallographic data is available for these SFAs. However, a clear preferred orientation can be identified from the difference in relative intensities of the corresponding diffraction peaks collected from the coatings and powdered form of SFAs (Fig. 3, a-f). Heat treatment induced structural reorganization of the coatings (Fig. 3, d-f, blue lines). Improvement in their crystallinity resulted in the reduction of the amorphous hump and appearance of additional diffraction peaks (Fig. 3, d-f, blue lines).

Additional investigation was performed in the case of the behenic acid (22C) coating, where the deposition was applied on a substrate pre-heated to 45 ​± ​2 ​°C in order to reduce the diffusional barrier during the crystal formation. These results proved that additional thermal energy given at the pre-deposition stage strongly affects the morphology, crystallinity and the roughness of the resulted coating (for additional information see Fig. S2).

We also studied the influence of the amount of the deposited SFAs on the properties of the obtained coatings. The experimental data fully support our previous findings: higher amount of the deposited SFAs of the group A caused crystal coarsening, while in the group B it led to the development of more prominent hierarchical structure, followed by change in the coatings' properties (for additional information see Fig. S3).

Having established the feasibility of the formation of superhydrophobic surface coatings comprised of fatty acids via thermal deposition, we further aimed at developing and studying a more facile, applicative and scalable approach utilizing a spraying technique. In contrast to thermal deposition, spraying offers a great simplicity and high versatility enabling fast application, while only basic facilities could be required.

**Fig. 4:**
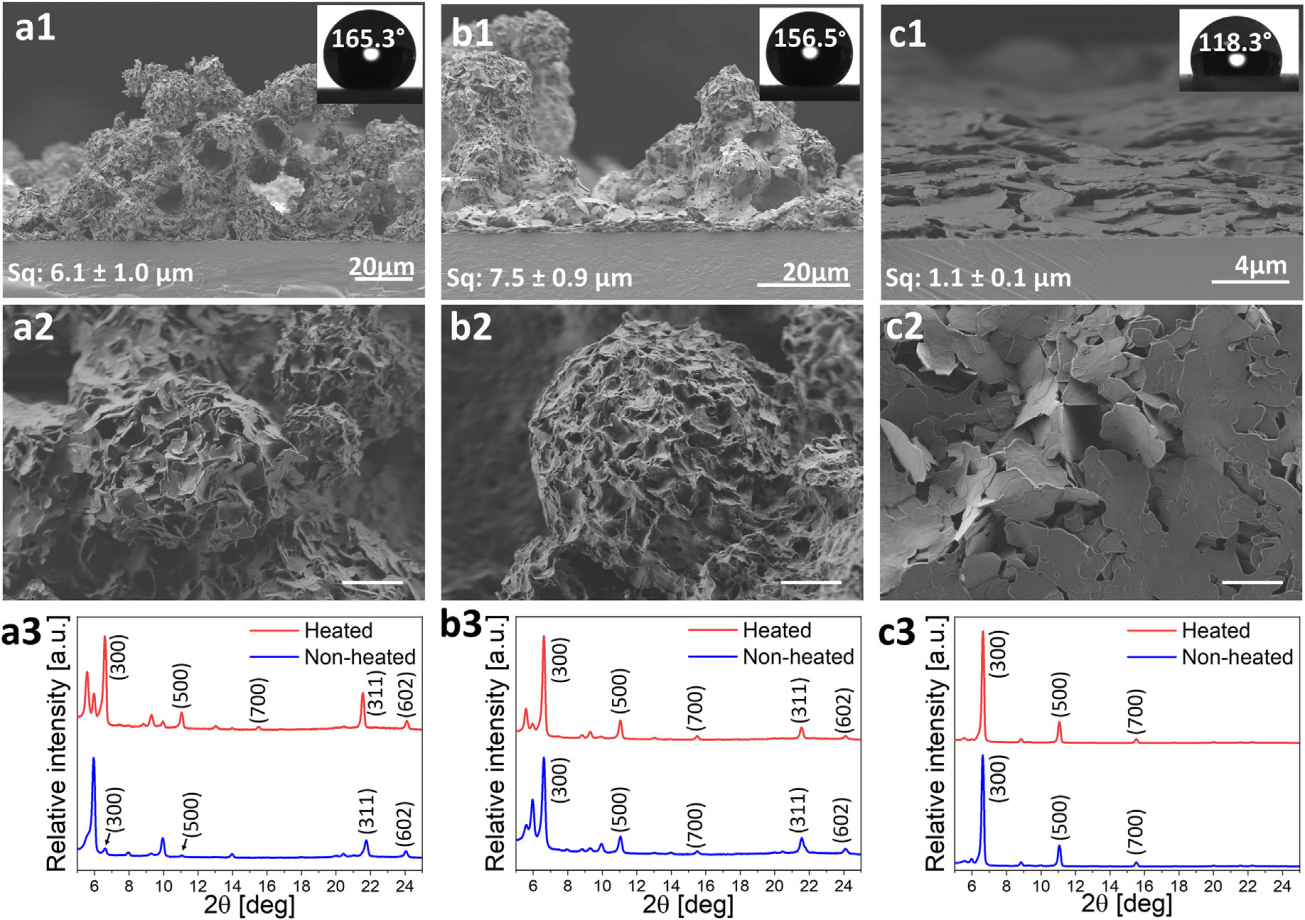
HR-SEM and XRD characterization of stearic acid (18C) spray coatings based on various solvents: a) diethyl ether, b) acetone, c) ethanol. a1-c1) cross-sectional views, respectively. Insets: water CA and roughness values. a2-c2) planar views of spray coatings, respectively. Scale bar is 4 ​μm a3-c3) XRD of spray coatings before and after heating, respectively (blue and red lines, respectively).

The first challenge was to define a suitable solvent allowing fabrication of the SFA-based spray solutions. We, therefore, studied the use of the three different solvents, namely ethanol, acetone and diethyl ether, in a test case of stearic acid (18C)-based spray. Resulted solutions were spray-deposited onto glass substrates and obtained coatings were characterized as for their morphology, wetting properties and surface roughness (Fig. 4).

Planar and cross-sectional imaging of the spray-deposited coatings revealed that in the case of diethyl ether- and acetone-based stearic acid (18C) coatings the surface is covered with sphere-shaped aggregates that are tens of microns in size and composed of smaller micron-sized crystals (Fig. 4, a1-b1 and a2-b2). Such hierarchical surface structure resulted in high roughness values of 6.1 ​± ​1 and 7.5 ​± ​0.9 ​μm, respectively. Both coatings demonstrated superhydrophobic behavior with water CA higher than 156° and CAH lower than 7° (see insets in Fig. 4, a1-b1). Reasonably poorer superhydrophobic performance with a CA of 118.3 ​± ​5.8°and a CAH of 11° was observed in the case of ethanol-based stearic acid (18C) spray coating, whose surface comprises closely packed platelet-like crystals with smooth surface (Fig. 4, c1-c2). As no hierarchical structure was formed, a rather low, as compared to those of acetone-based and diethyl ether-based coatings, surface roughness of 1.1 ​± ​0.1 ​μm was measured. Based on the obtained results, we could assume that the properties of the spray coatings depend on the volatility of the solvent. As the solvent is more volatile (in our case T_b, ethanol_ ​= ​78 ​°C ​> ​T_b, acetone_ ​= ​56 ​°C ​> ​T_b, diethyl ether_ ​= ​34 ​°C [58]) it evaporates faster so as facilitates the formation of SFAs crystal aggregates and their hierarchical organization, which in turns results in the improvement of the coatings' wetting properties. Among the three chosen solvents, diethyl ether-based stearic acid (18C) coating demonstrated the most optimal superhydrophobic properties.

The structure of the spray coatings was studied via XRD (Fig. 4, a3-c3, blue lines). A strong preferred orientation along the {h00} planes was observed in the case of stearic acid spray coating based on the EtOH solution as well as acetone solution (Fig. 4, a3-b3), as compared to the structure of its powdered form (Fig. 3b). The diffraction pattern collected from the diethyl ether-based coating (Fig. 4 c3) also differs from that of randomly-oriented stearic acid powder (Fig. 3, b) revealing the presence of preferred orientation, however, the {h00} planes are missing at their expected *2θ* values. Interestingly, unexpected diffraction peaks emerged in the case of acetone-based coating as well as diethyl-based coating (Fig. 4, c1-c2). These diffraction peaks are left-shifted relatively to (300) and (500) planes. Since the solvents are volatile and evaporate extremely fast when sprayed, new diffraction peaks may appear due to a change in *d*-spacings caused by residual strains in the crystal lattice. The calculation of such residual strains results in a value of 10.8–11.0%, which seems reasonable since the strain is distributed along the carbon chain of the molecule. In order to facilitate relaxation of strains, the coatings were heated for 24 ​h at 50 ​°C and characterized again by XRD (Fig. 4 c1-c3, red lines). The XRD of the heated coatings indeed showed that relative intensity of the additional peak decreased, while (300) plane intensity increased. This finding together with DCS analysis (Fig. S4) supports the assumption of the presence of strains in coatings' lattice due to extremely fast solvent evaporation.

We further used diethyl ether solvent in order to fabricate palmitic acid (16C) and arachidic acid (20C) spray coatings, since these fatty acids showed a good thermal stability when were deposited via thermal deposition method. As expected, the morphology, wetting properties, roughness, and crystallographic patterns of the obtained coatings were similar to those of the stearic acid coating deposited using the same solvent (for additional information see Fig. S5).

As compared to the corresponding thermally deposited coatings (Fig. 1, a-c), sprayed coatings surfaces are more heterogeneous with roughness values more than one order of magnitude larger than those of thermally deposited coatings (Fig. 2 c, Fig. 4 a1, Fig. S5). In this case, prominent hierarchical morphology is achieved due to the combination of the micro-roughness stemming from micro-sized spherical aggregates obtained as a result of a spraying process and their nano-roughness related to the self-assembly of the SFAs molecules. The latter results in an increased surface roughness and improves the superhydrophobic properties of the sprayed coatings. Indeed, the palmitic acid (16C) sprayed coating, for example, exhibited a CA>150°, while its thermally deposited counterpart demonstrated a CA lower than 140° (see Fig. S3). Overall, based on our observations, spray coating method was found to be easier in its application and provided better superhydrophobic characteristics to the coatings. Moreover, spray coatings exhibited an exceptional stability over at least 15 months at ambient conditions by maintaining their wetting properties and the roughness values, despite a slight structural evolution (see Fig. S6). Additionally, the wetting properties and overall morphology of both stearic (18C) and arachidic (20C) acid spray coatings that were preserved for a period of 7 days following their incubation in PBS at 37 ​°C, mimicking physiological conditions, were found to be stable. Arachidic acid (20C) coatings were also found to be stable under dynamic conditions at RT (for detailed information see Fig. S7). Adhesion tape-test showed a good peel resistance of the spray coatings along incisions and at their intersection. Even though following the adhesion tape test an overall coating thinning was observed, the CA was not significantly affected (see Fig. S8). However, these coatings did not exhibit abrasion resistance when an abrasive paper was applied on the surface.

Our previous studies demonstrated that in addition to the unique physical, wetting, and crystallographic properties of self-assembled wax coatings, they also exhibit exceptional anti-biofouling properties achieved via passive inhibition of bacterial adhesion to the surface [33,34]. The anti-biofouling and potential antimicrobial properties of the SFAs coatings, formed via spraying, were characterized using two common model bacteria, *E. coli* and *L. innocua*. The latter is a Gram-positive bacterium and a well-known indicator for the pathogenic *Listeria monocytogenes* [59]. While, *E. coli* is a Gram-negative bacterium, which is usually used as a model indicator bacterium since it is a typical inhabitant of the human intestinal tract [60].

**Fig. 5:**
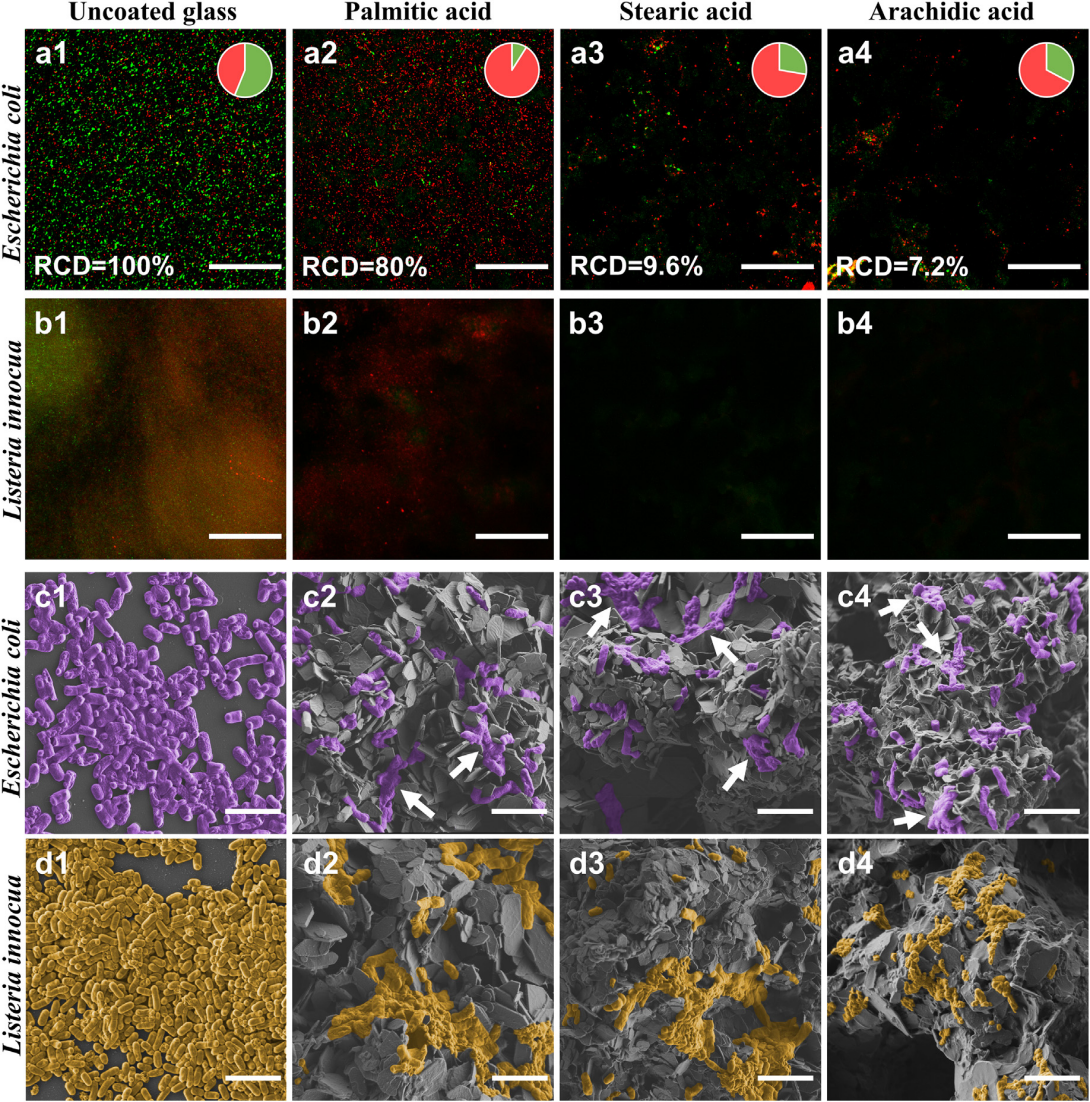
CLSM orthogonal projection images of the (a) *E. coli* and (b) *L. innocua* adhered onto surfaces after 48 ​h: 1) Uncoated glass (control), 2) Spray-coated palmitic acid (16C),3) Spray-coated stearic acid (18C), 4) Spray-coated arachidic acid (20C). Scale bar is 80 ​μm. Pie charts: relative bacteria cells density (RCD) per unit area, normalized per unit depth (cells μm^−3^); green and red sections represent live and dead cells, respectively. HR-SEM images of the (c) *E. coli* and (d) *L. innocua* adhered onto the surfaces: 1) Uncoated glass (control), 2) Spray-coated palmitic acid (16C), 3) Spray-coated stearic acid (18C), 4) Spray-coated arachidic acid (20C). Scale bar is 4 ​μm. Bacteria cells are false-colored to ease observation.

Bacterial attachment and viability onto the SFA coatings were characterized by live/dead staining followed by CLSM imaging analysis. Fig. 5, a-b shows representative three-dimensional orthogonal projection images of the coatings, depicting stained adhered bacterial cells where live/dead cells appear in green and red, respectively. Qualitatively, it is apparent for both species that the total number of adhered cells on all coated surfaces is reduced compared to the control uncoated substrate. For a more quantitative assessment, we used image analysis to calculate the relative bacterial cells surface density, termed as RCD (i.e., the number of cells per unit area, normalized per unit depth, presented relatively to control uncoated surface), and the respective values for *E. coli* are depicted in Fig. 5, a1-a4 pie charts. Note that in the case of *L. innocua* (Fig. 5, b1-b4), due to the dense bacteria population such analysis could not be performed as individual cells could not be easily distinguished. For palmitic acid (16C) coatings a small reduction in adherent cells is observed and the RCD value is decreased by 20% (Fig. 5, a1 vs. a2, b1 vs. b2). Stearic (18C) and arachidic (20C) acid coatings exhibit a profound anti-biofouling effect against both species, and *E. coli* adhesion is reduced by ∼90–93% (Fig. 5 a1 vs. a3 and a4, b1 vs. b3 and b4). Interestingly, in the case of stearic and arachidic acids no *L. innocua* cells were detected by means of CLSM imaging, see Fig. 5 b3-b4.

In addition to the reduction of adhered cells, the proportion of live cells decreases for all surfaces, indicating a biocidal effect of the coatings, particularly in the case of palmitic acid (16C) (Fig. 5 a1 vs. a2, b1 vs. b2 insets). Yet, it is important to note that in the case of stearic (18C) and arachidic (20C) acid coatings their strong anti-biofouling effect, resulting in significant reduction in bacteria count, may interfere with the assessment of their biocidal action (Fig. 5 a1 vs. a3-a4). This is further manifested in the case of *L. innocua* on stearic (18C) and arachidic (20C) acid coatings, where no fluorescence signal was detected in CLSM imaging.

Based on the CLSM studies we can suggest that there are two modes of action by which SFA surfaces function: anti-biofouling and biocidal. Such dual mode mechanism of action is reported in the literature for both synthetic [61] and natural superhydrophobic surfaces [62]. To elucidate the role of each of these effects, we studied the intrinsic antimicrobial activity of the corresponding fatty acids in powdered form (within bacteria media) and the results are summarized in Table S9. No inhibition effect of the powdered fatty acids was detected against *E. coli*; yet, for *L. innocua*, growth reduction of one order of magnitude was obtained for powdered palmitic (16C) and stearic (18C) acids (see Table S9). Surprisingly, powdered arachidic acid (20C) completely inhibited the growth of *L. innocua*. The intrinsic antibacterial properties of fatty acids are well documented, and unsaturated fatty acids are reported to exhibit superior potency against Gram-positive bacteria [44,45,63,64]. Yet, their exact mode of action is not fully understood, but may include disruption of membrane and electron transport chain of bacteria cells, inhibition of enzymes activity and protein synthesis. Thus, we suggest that the observed antibacterial properties of these coatings can be ascribed to the unique combination of their surface structure, which can induce cells rupture due to stretching and puncturing of the cell membrane [65,66], as well as the specific intrinsic biocidal activity of the used fatty acids. Therefore, the balance between these two effects may be contextual and species specific.

Next, we used HR-SEM as a complementary tool to qualitatively assess bacteria adhesion to the coatings. Given the highly hierarchical morphology of the coatings and their complex architecture (Fig. 4 a, Fig. S5 a, b), bacteria may not be fully exposed to the microscope laser beam using CLSM technique due to their adhesion to hidden locations on the surface and could result in their lower detection. Moreover, surface structure is one of the factors that may strongly affect the bacteria adhesion and their behavior on the surfaces [[67], [68], [69]]. Similar to the CLSM results, bacterial cells density on the SFA coatings is profoundly reduced in comparison to the uncoated substrates (Fig. 5 c1 vs. c2-c4 and d1 vs. d2-d4), which is attributed to the anti-biofouling effect of the coatings. Moreover, the electron micrographs reveal that the morphology of the SFA-adherent bacterial cells is distorted as compared to cells on the uncoated substrate, indicative of damaged cells [65,66,70,71]. In the case of *E. coli,* the cells are marked with white arrows in Fig. 5 c2-c4 and appear to be severely damaged, supporting the CLSM findings of reduced cells viability on the SFA coatings. In the case of *L. innocua*, it appears that most cells residing on the SFA coated surfaces have lost their integrity and are badly deformed and damaged (Fig. 5 d1 vs. d2-d4). These morphological changes corroborate well with the CLSM results and may be ascribed to the combined antibacterial functionality of these coatings, exerted by their hierarchical structure and intrinsic biocidal activity of the fatty acids (Fig. 5 b1-b4). Nonetheless, the balance between these two effects is likely to dictate their specific activity against different species, as manifested by superior activity of arachidic acid (20C) coatings against *L. innocua* cells.

Importantly, the antibacterial effect of the stearic (18C) and arachidic (20C) acid coatings was also observed after a prolonged incubation of 4–7 days in the respective bacterial suspensions. Fig. S10 shows that in the case of *L. innocua* (after 4 days), bacteria cells accumulate on the coated surfaces with time. Yet, these adhered cells are not viable, and their shape is highly distorted, as evidenced by both confocal and electron microscopy images (see Fig. S10 b and d). In the case of *E. coli* (after 7 days), surface coverage increases with time though to a lower extent in comparison to *L. innocua*; yet, sporadic viable cells are detected on the coated surfaces (see Fig. S10 a and c, and Fig. S11). These experiments may suggest that antibiofouling is the dominant mechanism in the case of *E. coli*, and biocidal effect is more prominent for *L. innocua*. For detailed information see Fig. S10 and Fig. S11.

To summarize, spray-deposited SFAs coatings demonstrate an effective prevention of *E. coli* and *L. innocua* adhesion over time in comparison to uncoated surfaces accompanied with additional strong biocidal effect against *L. innocua*, and a weaker biocidal effect against *E. coli*.

## 3 Conclusions

In this study we developed novel superhydrophobic coatings comprised of SFAs. We demonstrated the feasibility of the thermal deposition approach as a synthetic route to form superhydrophobic coatings with low CAH (<10°) comprised of SFAs with various chain lengths. By varying the molecule's length and deposited weight of selected SFA it is possible to control and tune the properties of the obtained coatings such as their CA, CAH, thickness and surface roughness. Even though the thermal treatment induced structural and morphological changes of the coatings, we showed that SFAs coatings maintain their superhydrophobic wetting properties after annealing up to 50 ​°C for at least 24 ​h.

Moreover, we developed an alternative facile spray coating method established via SFAs solvent-based formulations enabling a broad application of the coatings. The spray-coated surfaces demonstrated excellent superhydrophobicity, prominent hierarchical structure and high preferred orientation of the crystals. Most importantly, our preliminary results show that these coatings exhibit a unique combination of anti-biofouling and antibacterial properties against *E. coli* and *L. innocua*, used as relevant model bacteria. We suggest that the specific activity of the coatings against different bacteria stems from their complex hierarchal structure and the fatty acid's intrinsic biocidal effect. Thus, these coatings may be potentially tailored to exhibit a wide repertoire of desired antibacterial properties for different applications.

This new family of multifunctional coatings displays superior properties of superhydrophobicity as well as anti-biofouling and antimicrobial activities, while being safe and sustainable by design. We believe that this work provides a proof of concept for reliability of fatty acids to serve as functional antimicrobial coatings and further research may expand the range of properties that can be achieved for saturated fatty acid-based coatings.

## 4 Experimental section

### 4.1 Sample preparation

I. Thermal deposition of SFAs on glass substrates was performed using Moorfield Minilab coating system. The process was performed in a vacuum chamber at a pressure of ∼2 ​× ​10^−9^ [bar] by heating a crucible, which contains the coating material. Gradually increasing electrical current was applied in order to heat the crucible. The heat transforms the coating agent into a gas phase and the deposition is achieved when the fatty acid molecules contact the placed above substrate. The substrates were placed onto a rotating holder ∼10 ​cm above the crucible. After deposition the samples were stored in a freezer (−25 ​°C). An amount of 125 ​± ​1 ​mg of a fatty acid was used for the deposition, unless is specified differently.

The following saturated fatty acids were used as coating agents: Palmitic acid (98%, Acros Organics, Malaysia), Stearic acid (98.5%, Sigma, Switzerland), Arachidic acid (99%, Sigma-Aldrich, India), Behenic acid (96%, AA Blocks, USA), Lignoceric acid (96%, AA Blocks, USA) and Cerotic acid (97%, AA Blocks, USA).

II. Spray coating was performed using commercially available dye spray gun, connected to an air compressor. The same system setup was used to perform the deposition of all fatty acids. Palmitic acid (98%, Acros Organics, Malaysia), stearic acid (97%, Merck, Germany) and arachidic acid (99%, AA-Blocks, USA) were used for the coatings' preparation. Ethanol (ABS AR, Gadot, Israel), acetone (AR, Bio-Lab, Israel) or diethyl ether (stab. BHT, Bio-Lab, Israel) were used as solvents to prepare 20 ​mg/mL solutions. After the coatings were deposited, the samples were left overnight in vacuum oven at room temperature (RT) in order to remove any solvent residuals.

Thermal treatments of the coatings were performed using a Jeio Tech OV-11 oven at 50 ​°C. The oven was pre-heated and samples were inserted once the temperature is stable. The samples were heated for 24 ​h and after cooling to RT placed into the freezer (−25 ​°C) for storage.

*Characterization*: Morphology of the coatings was studied using 1 ​KV beam of high-resolution scanning electron microscope (HR-SEM) Zeiss Ultra Plus FEG-SEM. Prior the imaging, a conductive carbon coating was deposited onto the surface of the samples using a designated carbon coater. The same technique was used to image the cross-sections of the coatings. Prior to the coating deposition a scratch was implemented on the glass surface using a diamond scribe. Followed the deposition, samples were broken along the scratch and the exposed cross-sectional surface was observed using the HR-SEM.

Wetting properties of the coatings were characterized by CA and CAH measurements, which were performed using an Attension Theta Lite tensiometer and high-purity water or ethylene glycol (99.5%, Merck, Germany) droplets of 7 ​μL volume.

Roughness and coating thickness were measured using a dynamic confocal microscope (Leica DCM3D); data processing was performed using SensoMap Turbo software. The coating thickness was calculated as the difference in height between the lower and upper levels of a confocal profile measured after a scratch implementation on the coating using a 25G needle.

Structural characterization was performed using XRD measurements in a parallel beam theta-2theta mode using Cu anode sealed tube (Rigaku, SmartLab, X-ray Diffractometer). The preferred orientation degree, *η*, was calculated according to the March-Dollase method [72] and its extension [73]: $\eta {=\left[{\left(1-r\right)}^{3}/\left(1-r^{3}\right)\right]}^{0.5}∙100\%$. Parameter *r* is dependent on the angle, *α,* between the two compared planes, the preferred orientation plane and the reference plane and can be calculated as follows: $r={\left[\sin^{2}\;\alpha /\left({\left(k_{sample}/k_{powder}\right)}^{2/3}-\cos^{2}\;\alpha \right)\right]}^{1/3}$, where *k* is the ratio between intensities of the preferred orientation plane and the reference plane (calculated for the sample and randomly oriented powder).

Differential scanning calorimetry (DSC) was used to study the origin of the additional XRD diffraction peaks. Fatty acids were detached from the glass substrate of spray-coated samples and were examined using DSC (LabSys 131, SETARAM). A cyclic measurement was performed by heating the powder from 20 ​°C to 150 ​°C followed by cooling down to 20 ​°C at a rate of 0.17 ​°C sec^−1^.

The stability of the coatings under relevant physiological conditions was characterized following their incubation in phosphate buffered saline (PBS) at 37 ​°C and under orbital-horizontal shaking (∼40 ​rpm) at room temperature for 7 days.

Mechanical test of the coatings was based on ASTM D3359-17 [74]. The X-cut incisions were implemented using a laboratory scalpel. The test was performed using laboratory labelling tape which covered the incision and was pressed manually using a rubber patch. Dwell time of 90 ​s was utilized before the tape removal. The incisions area was observed before and after the tape test using reflected light at optical microscope (Olympus BX51). CA and roughness before and after the test were measured according to previously described methods.

*Bacterial cultures*: Gram-negative *Escherichia coli* (*E. coli*) ATCC 8739 was cultured in Luria Broth (LB) medium containing 10 ​g ​L^−1^ Bacto Tryptone (BD, USA), 5 ​g ​L^−1^ Bacto yeast extract (BD, USA) and 5 ​g ​L^−1^ sodium chloride (BioLab, Israel). LB agar plates for culturing were prepared by adding 18 ​g ​L^−1^ Bacto agar (BD, USA) to the LB medium.

Gram-positive *Listeria innocua* (*L. innocua*) *ATCC 33090* was cultured in Brain Herat (BH) medium containing 37 ​g ​L^−1^ ​BH Infusion (BD, USA). BH agar plates for culturing were prepared by adding 18 ​g ​L^−1^ Bacto agar to the BH medium.

The bacteria were cultured in the appropriate agar plate and stored at 4 ​°C. Next, one bacteria colony was incubated overnight in 4 ​mL liquid medium (LB or BH) at 37 ​°C under agitation (150 ​rpm) until the bacteria reached a stationary phase (∼10^9^ ​CFU ​mL^−1^). Then, the bacterial suspensions were diluted by 1:100 in liquid medium for further experiments.

*Characterization of bacterial adhesion onto SFA spray-coated surfaces*: SFA spray-coated surfaces were prepared as described in “Samples preparation” section using 10 ​mm round cover glass for slides as a substrate. The coated samples were UV sterilized prior to use. The samples placed into 6-well plates and incubated with 4 ​mL of the respective bacterial suspension at 37 ​°C for 48 ​± ​2 ​h. The samples were removed from the suspension and bacteria viability was studied by using a live/dead BacLight viability kit, where a 0.3% solution concentration (0.15% concentration of each reagent) was used. In the case of longer incubation, the medium was replaced every 48 ​h. Subsequently, for three-dimensional image projection of the samples a confocal laser scanning microscope (CLSM), Zeiss LSM 510 META, was used. Combinations of 488 ​nm and 561 ​nm laser lines were used for the excitation of live bacteria and dead bacteria, respectively. Quantitation of adhered live/dead bacteria on the surface based on CLSM fluorescent images was performed using Spots analysis in Imaris 9.3.1 software. The values were normalized per depth unit to neutralize thickness difference of the coatings.

HR-SEM micrographs were obtained after bacteria were fixed on the surfaces using a glutaraldehyde solution (2% in 0.1 ​M normal saline) followed by dehydration through an ethanol series. Then, the samples were dried under vacuum overnight and sputtered with a conductive carbon coating.

### 4.2 Antimicrobial studies

The antimicrobial properties of the different SFA powders were evaluated by in liquid medium via the drop-plate method. One bacteria colony (*E. coli* or *L. innocua*) was incubated overnight in 4 ​mL Nutrient Broth (NB) liquid medium (Sigma Aldrich, Israel) at 37 ​°C under agitation (150 ​rpm) until the bacteria reached a stationary phase (∼10^9^ ​CFU ​mL^−1^). Then, the overnight bacteria culture was diluted in fresh NB medium (∼10^7^ ​CFU ​mL^−1^) and incubated for an additional 2 ​h to achieve a logarithmic culture. Next, the logarithmic culture was diluted to 10^4^ ​CFU ​mL^−1^ in 1:100 NB medium. 1 ​mL of the diluted bacterial suspension was incubated with 50 ​mg of different SFA powders at 37 ​°C for 24 ​h in 24-well-plates under agitation (100 ​rpm). The cultures were decimally diluted and 10 ​μL drops were transferred onto NB solid agar substrate. The colonies were counted after 24 ​h incubation at 37 ​°C.

## Declaration of competing interest

The authors declare that they have no known competing financial interests or personal relationships that could have appeared to influence the work reported in this paper.

## Data Availability

Data will be made available on request.

## Appendix A Supplementary data

SFA properties table; characterization of thermally deposited behenic acid on heated substrate (HR-SEM, CA, roughness, XRD); weight-dependent coatings characterization (HR-SEM, CA, roughness, XRD); DSC of stearic acid after different solvent-based spray processing; sprayed SFA coatings characterization (HR-SEM, CA, roughness, XRD, DSC); 15-months spray coatings stability data (HR-SEM, CA, CAH, roughness, XRD); adhesion tests of spray coatings (images, optical microscope images, CA, roughness); bacteria count after incubation with powdered SFAs.
