# Supplementary material for: Self-assembled fatty acid crystalline coatings display superhydrophobic antimicrobial properties
Source: Mater Today Bio. 2022 Dec 8;18:100516. doi: 10.1016/j.mtbio.2022.100516 (PMC9771733; doi:10.1016/j.mtbio.2022.100516)
Supplement: Multimedia component 1 [file mmc1.docx]

Self-Assembled Fatty Acid Crystalline Coatings Display Non-Toxic Superhydrophobic Antimicrobial Properties

Elena Prudnikov ^a^, Iryna Polishchuk ^a^, Andy Sand ^b^, Hanan Abu Hamad ^b^, Naama Massad-Ivanir ^b^, Ester Segal ^b*^ and Boaz Pokroy ^a*^

^a*^ Department of Materials Science and Engineering, Technion − Israel Institute of Technology, 3200003 Haifa, Israel. E-mail: bpokroy@tx.technion.ac.il; Tel: +972-4-829-4584

^b*^ Faculty of Biotechnology and Food Engineering, Technion − Israel Institute of Technology, 3200003 Haifa, Israel

**KEYWORDS:** superhydrophobic coatings, saturated fatty acids, thermal deposition, spray coating, antimicrobial, anti-biofouling, biocidal

**Supporting Information**

**Table S1:** Common names and physical properties of the tested saturated fatty acids.

| Fatty acid name | No. of carbons | Melting temperature [°C]^[1]^ |
| --- | --- | --- |
| Palmitic acid | 16 | 62.5 – 63.1 |
| Stearic acid | 18 | 67 – 69.6 |
| Arachidic acid | 20 | 75.3 – 75.4 |
| Behenic acid | 22 | 79.9 – 80.0 |
| Lignoceric acid | 24 | 75 – 83 |
| Cerotic acid | 26 | 87 – 88 |


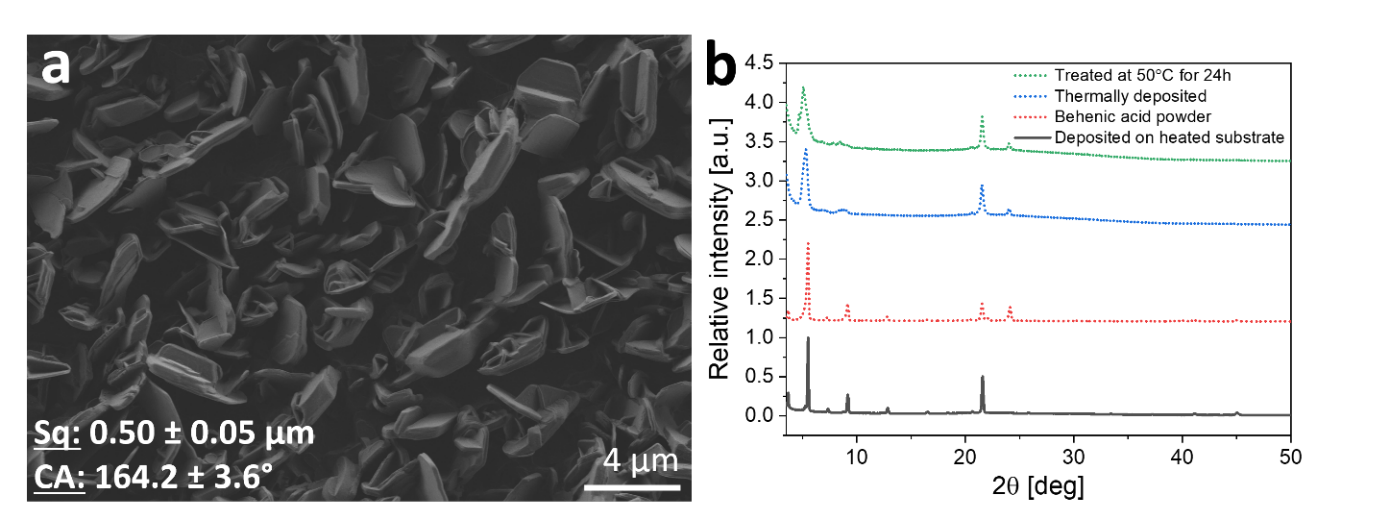
To investigate the significant morphological difference of the coatings from group A and group B, deposition of behenic acid (22C) was performed during a substrate heating to 45±2ºC to reduce the diffusional barrier and **Figure S2** presents representative electron micrograph and XRD diffractograms.

**Figure S2:** Characterization of deposited behenic acid on heated glass substrate: a: HR-SEM micrograph of obtained surface, roughness, and water contact angle measurements. b: XRD pattern of deposited coating on heated glass substrate (solid line) compared to previously showed powder, coating and heated coating diffractions (dashed lines).

Interestingly, when the deposition was performed upon heating, completely different morphology was obtained: the grown crystals are similar in shape to the crystals of fatty acids from group A, but larger in size (**Figure 1 a, b, c, Figure S2 a**). Moreover, the crystals also differ in their shape from the crystals of behenic acid coating that was heated at 50°C for 24 h after the deposition (**Figure 1d** - inset). This experiment shows that not only crystals size can be controlled if the deposition performed on heated substrate, but also their overall shape. Comparing to behenic acid deposited on non-heated substrate, the contact angle was not changed significantly (164.2±3.6° vs. 168.4±4.4°), but the roughness was changed from 1.25 µm to 0.5 µm on heated substrate, a value which is typical to the roughness values of shorter fatty acid coatings (0.30-0.34 µm) (**Figure 2c**, **Figure S2** **a**). The XRD pattern also differs from the XRD of behenic acid deposited on unheated substrate as well as from XRD of thermally treated coating: the amorphous hump disappeared as well as the peak at ~24.1° that exist in powder and coatings diffractions (**Figure S2** **b**). This supports the explanation that the crystals shape changed due to improved crystallinity of the deposited material; an addition of thermal energy can compensate the missing energy for more effective crystallization of longer molecules with higher diffusional barrier for crystallization. However, it is important to note that the heating have to be implemented at the deposition step; when similar temperature was used for thermal treatment (50°C for 24h) the required change was not obtained; the morphological and structural changes of already deposited coating require more energy for molecules reorganization than the energy required at the step of deposition from vapor state.

To this end, different amounts of palmitic acid (16C) and cerotic acid (26C) were deposited on glass substrates for better understanding of film formation process (**Figure S3**). For palmitic acid (16C), a decrease of deposited weight down to 27 mg resulted with different morphology relatively to thicker coatings: smooth surface with structured areas where a crystals growth is initiated was formed (**Figure S3 a**). The same unstructured coating is observed in cross section images of deposited SFAs, mainly in palmitic acid (16C) (**Figure 1a**). This initial wetting surface coating exist in all samples and probably provides the base for crystals growth that are formed from afterwards deposited SFAs. As a result of flat non-structured surface, lower ethylene glycol CA (72.0°) and lower roughness (0.04 µm) relative to 126 mg deposited coating (CA 131.3° and Sq 0.34 µm) were obtained. The difference of the thinner coating can be also seen from the XRD pattern: the intensity of preferred (311) peak of the thicker coatings is significantly lower for the 27 mg deposited coating, indicating the {h00} preferred orientation of the initial wetting layer (**Figure S3 g**). Interestingly, for deposited 451 mg coating of palmitic acid the CA of ethylene glycol decreased relatively to 126 mg coating down to 93.7°, while the roughness increased up to 0.78 µm. Based on the HR-SEM micrograph, the developed crystals are coarser; that caused to an increase of the solid-liquid interface and as a result the CA decreased (**Figure S3 c**).

Oppositely to palmitic acid (16C), even a deposition of 5-fold smaller amount (25 mg) of cerotic acid (26C) resulted with characteristic surface morphology, similarly to the observed crystals when higher amount was deposited (**Figure S3 d, e, f**). For this case, the more cerotic acid (26C) was deposited, the more prominent hierarchical structure was developed and correspondingly, higher ethylene glycol contact angle and roughness values were achieved (**Figure S3** insets in **d, e, f**). The XRD patterns of cerotic acid coatings remained similar for different deposited weights; the two main peaks exist for all the samples and a minor decrease of the amorphous hump of 450 mg deposited sample indicate higher crystallinity of that coating (**Figure S3 h**).

Overall, deposition of longer SFAs can provide better superhydrophobicity and more prominent hierarchical structure when smaller material amount is used relative to shorter SFAs.


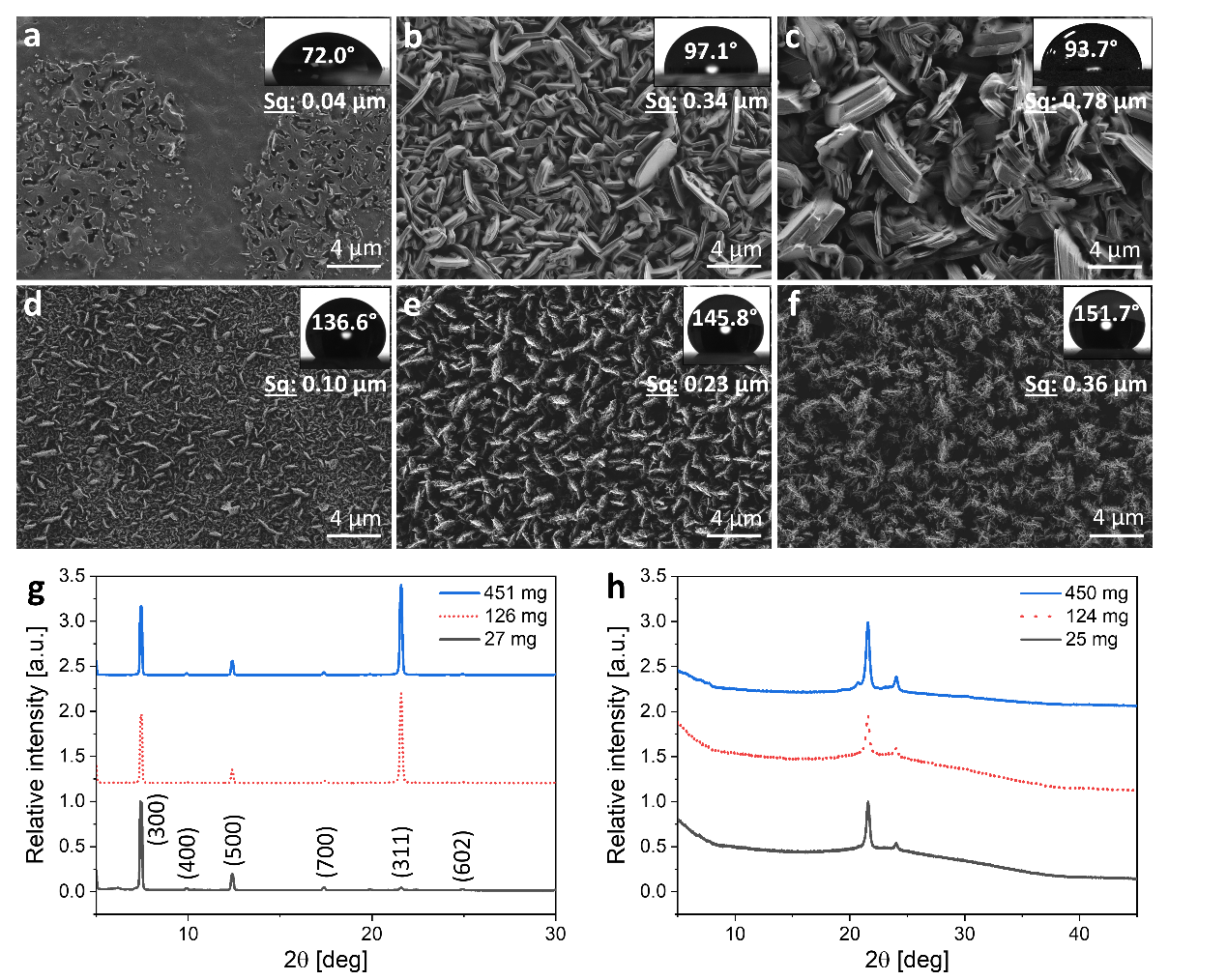


**Figure S3:** Characterization of coatings of different deposited weight of palmitic acid and cerotic acid. a-f) HR-SEM micrographs, insets: contact angles of ethylene glycol and roughness values. a) Palmitic acid, 27 mg, b) Palmitic acid 126 mg, c) Palmitic acid 451 mg, d) Cerotic acid 25 mg, e) Cerotic acid 124 mg, f) Cerotic acid 450 mg. g) XRD of palmitic acid different deposited weight coatings, h) XRD of different deposited weight coatings of cerotic acid.

The coatings were characterized by DSC and the results are presented in **Figure S4**. The DSC of unprocessed stearic acid, acetone- and ethanol-based sprayed stearic acid show curves that comprise single endothermal and exothermal peaks at the heating and cooling curves respectively, proving the conclusion from heat-treated samples XRD that the left-shifted unexpected peaks origin is in the lattice distortion but not in impurities (**Figure S4 a, c, d**). The heating curve of diethyl ether-based spray contains additional endothermal peaks that indicate a contamination of the sample that could also contribute to the XRD pattern (**Figure S4 b**, **Figure 4, a3-c3**). However, since similar unexpected peaks appeared at both acetone- and even more significantly in diethyl ether-based sprays, and thermal treatment caused to decrease of these peaks’ intensity while the main (300) peak intensity increased, it could be concluded that the main contribution to these peaks’ appearance is of the lattice distortion.


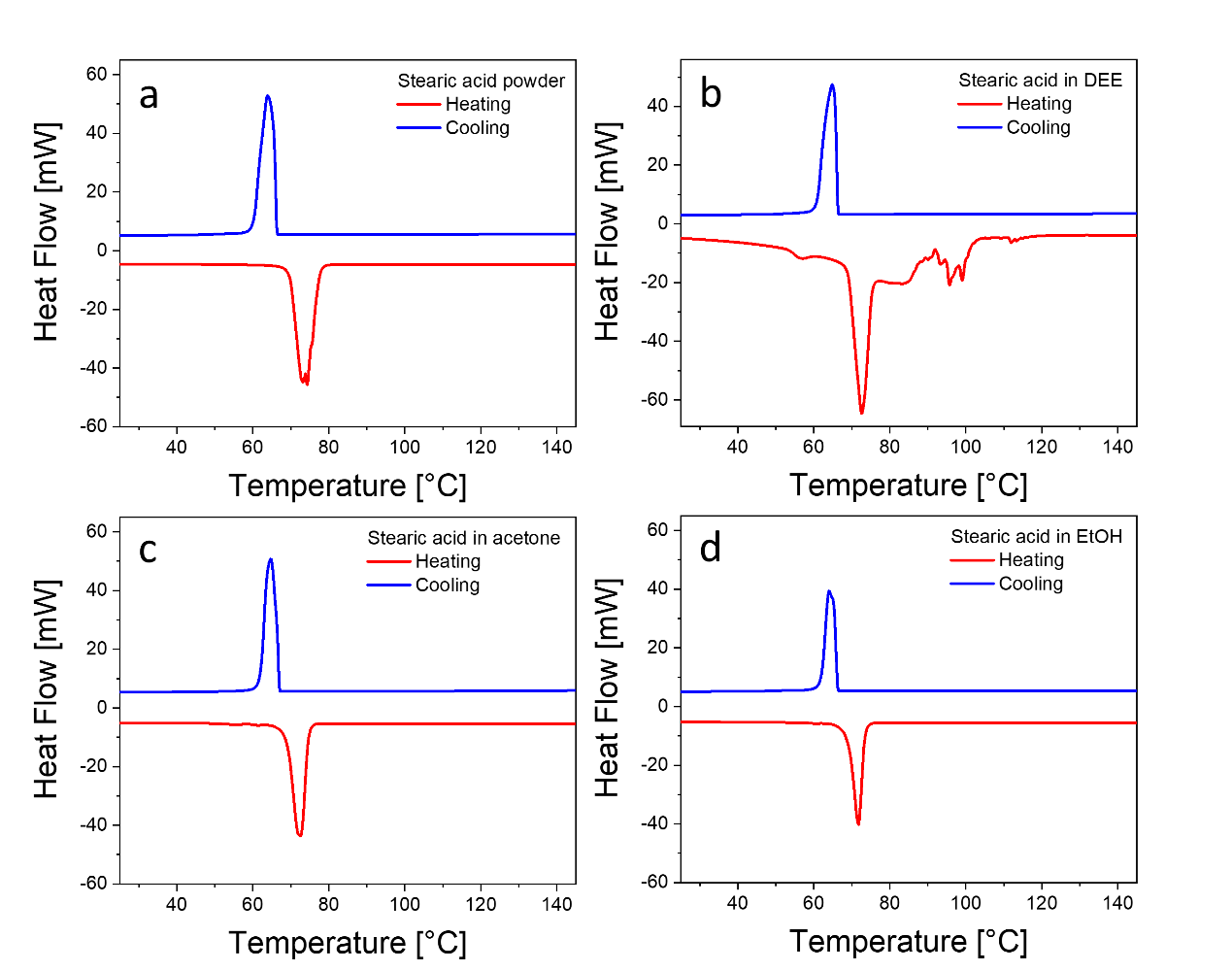


**Figure S4:** DSC curves of non-processed powder and of different solvent-based sprayed stearic acid (18C). a. non-processed powder, b. diethyl-ether-sprayed, c. acetone-sprayed, c. ethanol-sprayed.

Characterization of palmitic acid and arachidic acid diethyl ether based sprayed coatings:

**Figure S5 a-b** shows the morphology of palmitic and arachidic acids diethyl ether-based sprayed surfaces, which are very similar to the morphology of stearic acid diethyl ether-based sprayed sample (**Figure 4a**). Their surface is covered with aggregates of crystals, which contribute to creating a high surface roughness several microns, respectively, as well as CAs higher than 159° and CAH lower than 6º for both coatings (**Figure S5 a1, b1**). Similar phenomena of unexpected XRD left-shifted peak that was observed for deposited stearic acid (**Figure 4 c1**) can be can be observed in **Figure S5 e**. DSC results of unprocessed palmitic acid (**Figure S5 c1, c2, d1, d2**) show endothermal and exothermal single peaks during the heating and the cooling processes, respectively, with few endothermal peaks during the heating of the processed sample, similarly to DSC of diethyl ether-processed stearic acid (**Figure S4 b**). Two additional endothermal peaks at lower temperature than the main peak of arachidic acid powder appear **(Figure S5 d1**) and may indicate residual impurities in that powder with no relation to spray processing. These peaks did not appear at cooling process. Cooling DSC curves of all SFAs remained clear, as the cooling curve of stearic acid.


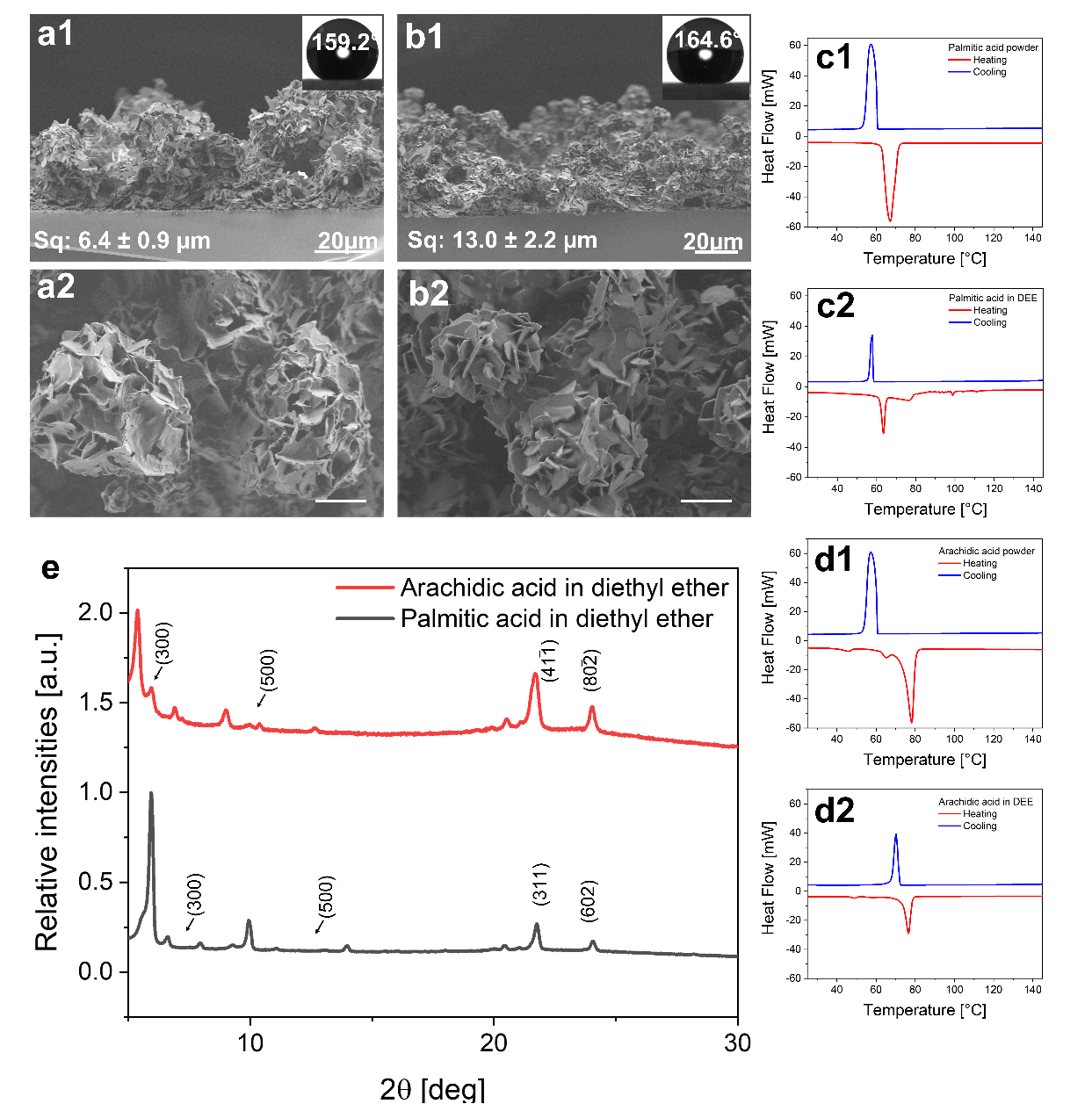


**Figure S5:** Characterization of a. palmitic acid and b. arachidic acid diethyl ether-based coatings: a1-b1 – HR-SEM cross-sectional views. Insets: water CA and roughness values. a2 – b2 – planar views of spray coatings, scale bar is 4 µm, c-d - DSC measurements of non-processed powders and diethyl ether-based sprayed SFAs: c1 – palmitic acid powder, c2 – sprayed palmitic acid, d1 – arachidic acid powder, d2 – sprayed arachidic acid. e – XRD of deposited coatings.

To study the stability of different spray coatings, their properties were evaluated after storage of 15 months at ambient conditions, and the results are presented in **Figure S6.**
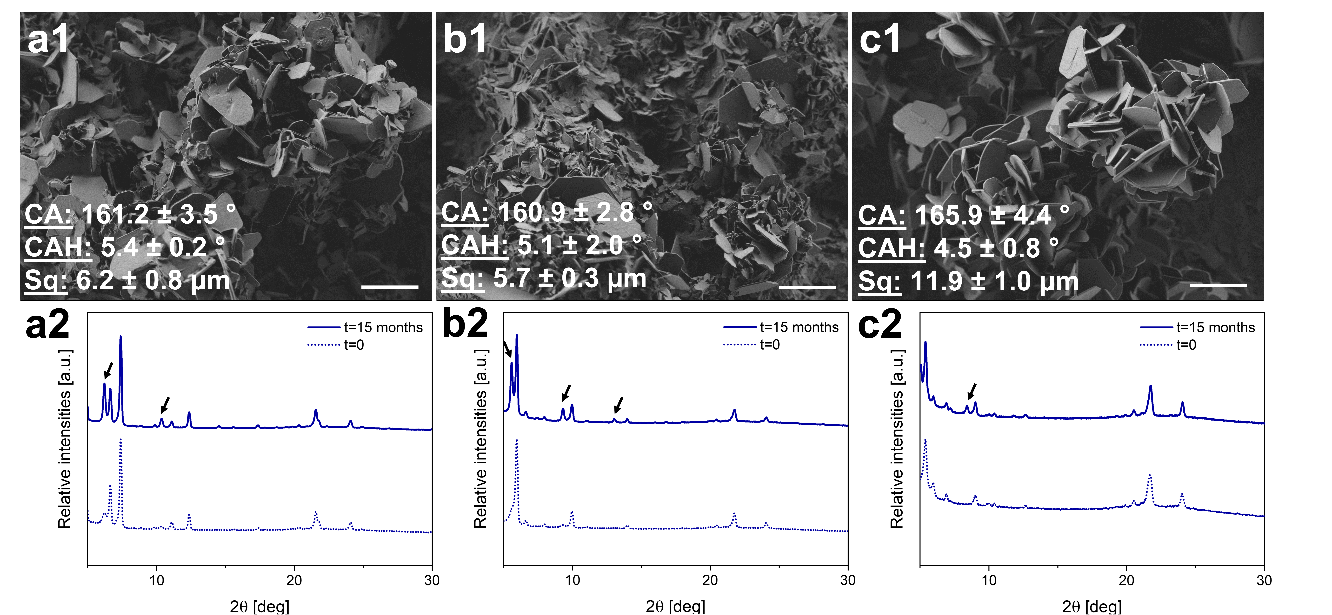


**Figure S6:** Characterization of diethyl ether-based sprayed coatings after 15-months storage at ambient conditions: a – palmitic acid, b – stearic acid, c – arachidic acid. a1-c1 – HR-SEM planar views of spray coatings, scale bar is 8 µm. Insets: water CA, CAH and roughness values. a2-c2 – XRD of deposited coatings at t=0 and t=15 months.

HR-SEM images in **Figure S6 a1-c1** show crystal growth of fatty acid plates over time, relatively to the images obtained at t=0, shown in **Figure 4 a2**, **Figure S5 a2, b2**. New peaks appearance in XRD in **Figure S6 a2-c2** also indicate structural reorganization of the fatty acids, probably due to strain relaxation. However, the wetting properties and overall roughness is maintained despite a slight structural evolution (**Figure S6, a1-c1** insets).

The stability of the coatings under physiological conditions (incubation in PBS at 37°C) was evaluated after 7 days as well as following and under dynamic conditions at RT to evaluate the potential of biomedical applications in wet environment. The results are shown in **Figure S7**:


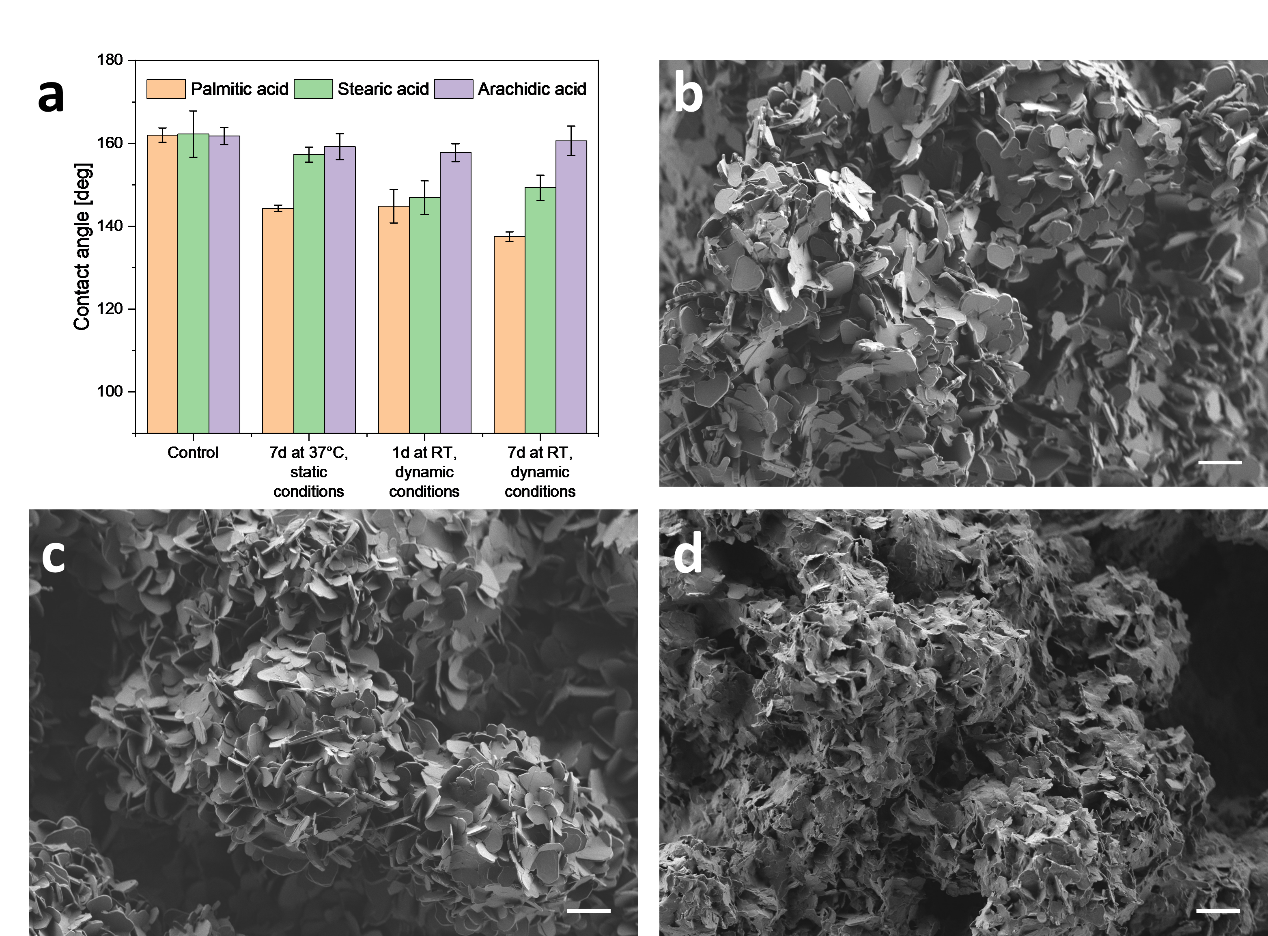


**Figure S7:** Sprayed SFA coating properties after incubation in PBS under different conditions: a. contact angle of the post-immersed coatings after washing and drying. HR-SEM images of the coatings after immersion at RT under dynamic conditions: b. palmitic acid (16C), c. stearic acid (18C), d. arachidic acid (20C).

**Figure S7** **a** show excellent stability of the contact angle of arachidic acid (20C) coating under all tested conditions: 7 days in PBS at 37°C under static conditions and up to 7 days in PBS at RT under dynamic conditions; the measured contact angle after sample wash and dry remained similar to the contact angle of untreated control sample. Stearic acid (18C) coating maintained it`s superhydrophobicity after 7 days incubation at 37°C under static conditions, but slightly decreased contact angle was measured after immersion under dynamic conditions. The contact angle of palmitic acid (16C) coating decreased below 150° after all tested conditions, but remained hydrophobic. The overall coatings morphology remained unchanged after immersion into PBS for 7 days under dynamic conditions despite of decrease of the contact angle. (**Figure S7** **b-c**). Based on these results, the coatings can be used under the tested conditions, while the composition can be chosen according to the requirements of actual application.

To test the adhesion of sprayed fatty acid coatings, adhesion tape test, based on ASTM D3359-17, was implemented. The results are shown in **Figure S8:**


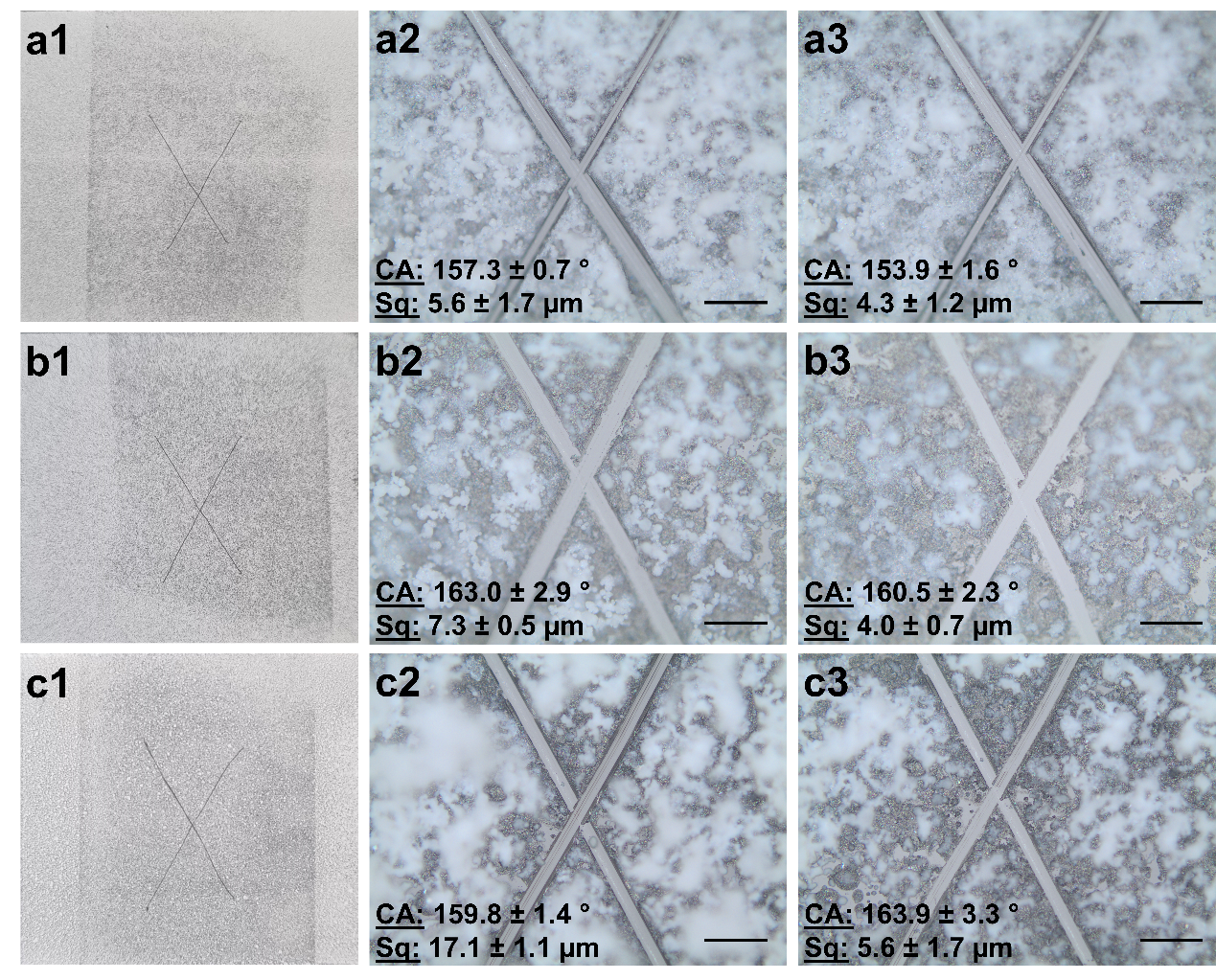


**Figure S8:** Images of a. palmitic acid, b. stearic acid and c. arachidic acid coatings at tape test procedure. a1-c1: images of the implemented X-cut and the area after tape removal. a2-c2: optical microscope images of the scratch area before the tape test. Insets: CA and roughness values. a3-c3: optical microscope images of the scratch area after the tape test. Insets: CA and roughness values. scalebar is 100 µm.

**Figure S8 a1-c1** show the incisions area, where the tape test was implemented. Coating thinning can be easily recognized by its increased transparency (the samples are placed on a black background). This finding is supported by optical microscope images before the test (**Figure S8 a2-c2**) and after the test (**Figure S8 a3-c3**) by reduced amount of fatty acid aggregates that are recognized on the post-test images, in accordance with the reduced roughness values on the post-test coatings (**Figure S8** insets). Despite the coating thinning, the X-cut appearance remained similar after the test and no coating peeling was recognized. Importantly, the CA of the coatings did not change significantly after the tape test, maintaining the superhydrophobicity.

To study the contribution of surface chemistry to the antimicrobial properties of the spray-coated surfaces we eliminated the surface morphology factor by using SFA powders. SFA powders were added to both bacteria culture, incubated for ~24h at 37°C and plated on NB agar for ~24h at 37°C. The results are summarized in the **Table S9**:

**Table S9:** E. coli and L. innocua count after the incubation with SFA powders in liquid media for 24h at 37ᵒC estimated by the drop-plate method.

| Sample | *E. coli* [CFU mL^-1^*] | *L. innocua* [CFU mL^-1^] |
| --- | --- | --- |
| Control | $1.2\pm0.2\cdot{10}^{8}$ | $2.4\pm0.2\cdot{10}^{5}$ |
| Palmitic acid (16C) | $1.4\pm0.6\cdot{10}^{8}$ | $1.7\pm0.5\cdot{10}^{4}$ |
| Stearic acid (18C) | $1.8\pm0.05\cdot{10}^{8}$ | $1.9\pm0.5\cdot{10}^{4}$ |
| Arachidic acid (20C) | $9.6\pm3.2\cdot{10}^{7}$ | $0$ |

*CFU – Colony-forming unit

**Table S9** shows the effect of added powders of SFAs on bacterial growth. The results show that the tested SFAs have no inhibiting effect on Gram-negative *E. coli* growth; same order of magnitude of colonies number was obtained when control sample and samples that were incubated with SFAs presence were cultured. Following this result, it can be concluded that the surface morphology has the main contribution to the antimicrobial effect of the sprayed SFAs coatings. However, the same SFAs do have growth inhibition effect on Gram-positive *L. innocua*: one order of magnitude growth inhibition was seen after addition of palmitic acid (16C) and stearic acid (18C) and full growth inhibition was achieved when arachidic acid (20C) powder was added to the samples. This bacterial growth inhibition effect probably contributes to increased dead\live cells ratio that was seen on CLSM images and to the cells morphology change observed on HR-SEM (**Figure 5**). However, it is important to notice that when the fatty acid is crystallized into micron-size crystals during the deposition process, the chemical effect may be decreased due to lower availability of the SFA relatively to powdered SFA.

The effect of time on the antibiofouling activity of the coatings was evaluated following their incubation in bacterial suspensions (see details in the Experimental section, note that the medium was replaced with fresh medium every 48 h).


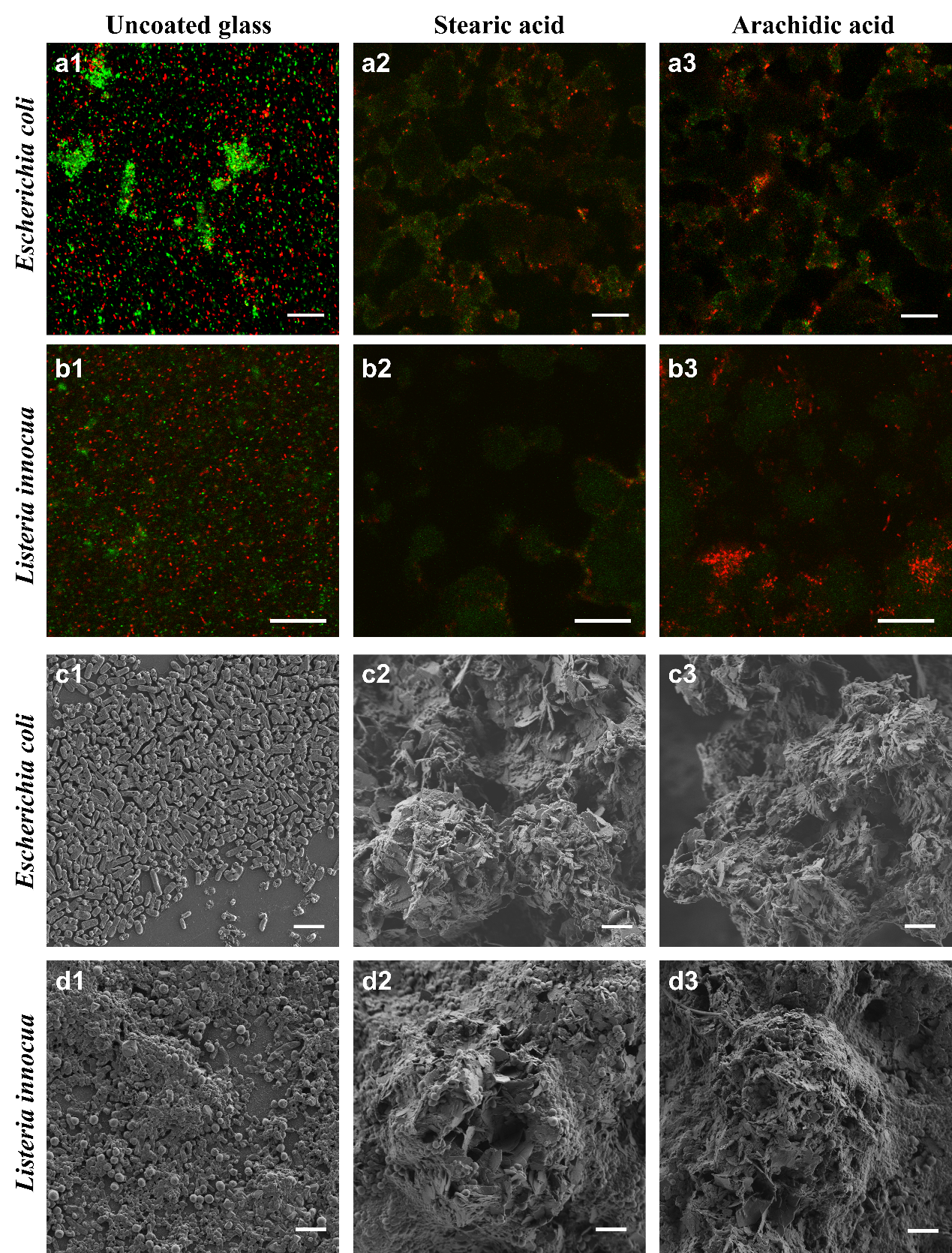


**Figure S10:** CLSM images of the spray-coated surfaces following their incubation in bacterial suspensions (a) E. coli for 7 days and (b) L. innocua for 4 days. (1) Uncoated glass (control), (2) Spray-coated stearic acid (18C), (3) Spray-coated arachidic acid (20C). Scale bar is 80 µm. HR-SEM images of the coated surfaces following their incubation in (c) E. coli suspension for after 7 days and (d) L. innocua suspension for 4 days. (1) Uncoated glass (control), (2) Spray-coated stearic acid (18C), (3) Spray-coated arachidic acid (20C). Scale bar is 4 µm.


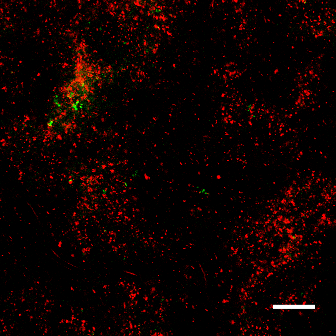


**Figure S11**: CLSM image of E. coli on palmitic acid (16C) coating after 5 days incubation.

The CLSM images reveal that bacteria accumulate with time on the surface of fatty acid aggregates (see **Figure S10 a2-a3** and **b2-b3** relatively to **Figure 5** **a3-a4** and **b3-b4)**; yet, most of the adhered bacteria appear to be dead. Importantly, live bacteria amount exceeds the dead bacteria amount on the uncoated glass surfaces (**Figure S10 a1**, **b1**). It is important to note that the coatings did not maintain their stiffness and adhesion to substrate when incubated with *L. innocua* for more than 4 days: the characteristic morphology of spherical aggregates was lost, and the material could be easily removed from the substrate. Similar deterioration of the palmitic acid (16C) coating was observed following 5 days of incubation with *E. coli*: the characteristic coating morphology could not be recognized under the microscope, but most of the recognized bacteria cells on the surface were dead (**Figure S11**). However, incubation with *E. coli* for at least 7 days did not damage the characteristic morphology of stearic (18C) and arachidic (20C) acid coatings.

HR-SEM images support the CLSM results: indeed, more adhered *E. coli* bacteria could be found on the coatings surface relatively to the observed bacteria after 48h, while similarly to the HR-SEM images after 48h, the bacteria morphology indicates that most of the cells are dead (**Figure S10 c2-c3**, **Figure 5** **c3-c4**). HR-SEM images of coatings incubated with *L. innocua* show much higher coverage of the surface by bacteria cells, but also in that case the original morphology of viable cells could not be recognized (**Figure S10 d2-d3**, **Figure 5** **d3-d4**). That observation may indicate an increased bactericidal effect of SFAs coatings on *L. innocua* (correspondingly the observed effect of powdered SFAs on *L. innocua*, **Table S9**) in comparison to higher antibiofouling effect of the SFAs on *E. coli*.

**REFERENCES**

[1] E. Tvrzicka, L.-S. Kremmyda, B. Stankova, A. Zak, *Biomed. Pap.* **2011**, *155*, 117.
